# Automation and Active Learning for the Multi‐Objective Optimization of Antibody Formulations

**DOI:** 10.1002/advs.76551

**Published:** 2026-07-13

**Authors:** D. Christopher Radford, Matthew Tamasi, Elena Di Mare, Adam J. Gormley

**Affiliations:** ^1^ Department of Biomedical Engineering Rutgers The State University of New Jersey Piscataway New Jersey USA

**Keywords:** bayesian optimization, biologics, GRAS excipients, machine learning, protein formulation

## Abstract

Over the last forty years, monoclonal antibodies have become increasingly important therapeutic agents, with most manufactured as preformulated solutions. However, bioformulation of complex proteins is a difficult engineering challenge; formulations must be tailored to individual therapies, necessitating time‐ and material‐intensive campaigns to select combinations of excipients to simultaneously optimize various design criteria. These additives complicate formulation design with unintuitive and non‐linear relationships, creating a vast multidimensional design space that is intrinsically difficult to optimize using traditional techniques. To address this challenge, we investigated a high‐throughput discovery pipeline using machine learning to model and predict the effects of GRAS excipients on formulation behavior of a model antibody. This was supported by automation‐assisted “on‐demand” formulation to produce dozens of uniquely formulated antibody solutions for downstream evaluation and biophysical characterization. This pipeline was then integrated into an iterative closed‐loop cycle of automated Design‐Build‐Test‐Learn (DBTL), where new rounds of experiments are designed by the model. The process yielded both improved formulations and accurate predictive models of formulation behavior across multiple target objectives (melting temperature, diffusivity, and high‐concentration viscosity). This validates the utility of this technique to both map the underlying property‐function landscape and effectively guide formulation development while balancing multiple competing design requirements.

## Graphical abstract

Successful antibody formulation necessitates balancing factors such as thermal stability, colloidal stability, and viscosity across a vast excipient design space. This work integrates robotic liquid handling, high‐throughput biophysical characterization, and multi‐objective Bayesian optimization in an iterative closed‐loop Design‐Build‐Test‐Learn cycle. Two active‐learning rounds yield improved formulations and accurate predictive models, while explainable AI uncovers mechanistic trade‐offs between excipients, guiding formulation design.

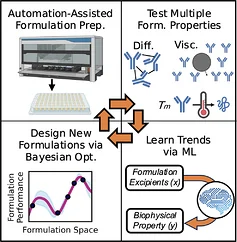

## 1 Introduction

Antibody‐based therapeutics are an increasingly important part of the clinical armamentarium [1]. The first monoclonal antibody treatment (Muromonab) was approved by the FDA for the US market in 1986 [2]. Since then, over 200 antibody‐based therapies have been approved globally and an additional 178 are currently in late‐stage clinical trials as of December 2024 [3]. These represent numerous key breakthroughs and serve as frontline therapies in the fields of oncology, immunology, and infectious disease [4, 5]. Antibody‐based therapies are typically administered via intravenous or subcutaneous routes [6]. As such, the majority are manufactured and provided to patients or providers as pre‐formulated solutions rather than in solid dosage forms [7]. However, the process of formulating biologics such as antibodies presents numerous challenges. As large, complex macromolecules, antibodies are prone to denaturation and aggregation [8]. These leave a fraction of the product in an inactive form, reducing the effective dose the patient receives. This can also alter epitope presentation, presenting an immunogenic risk to the patient [9]. As such, formulated antibodies must exhibit strong thermal and colloidal stability to ensure the active agent maintains a reasonable shelf life. Additional considerations might also arise based on the delivery route. For example, subcutaneous administrations inherently limit dosage volume, necessitating formulation at high antibody concentrations (typically >100 mg/mL) to achieve the requisite therapeutic dose [10]. At these concentrations, where amphiphilic proteins now occupy substantial volume, intermolecular interactions dominate and drive exponential increases in solution viscosity and poor injectability [11, 12, 13]. This necessitates a formulation strategy that explicitly accounts for viscosity properties of the solution.

Clinical antibody formulations typically rely on combinations of so called “Generally Recognized as Safe” (GRAS) excipients to achieve favorable properties while ensuring safety [7]. This class of excipients includes components used to maintain pH, osmolarity, viscosity, and solubility [14]. Clinical formulations can contain numerous components such as buffering agents (sodium citrate, sodium phosphate, sodium acetate), salts (typically sodium chloride), sugars (sucrose, trehalose, mannitol), amino acids (histidine, methionine, proline, arginine, lysine) and surfactants (polysorbate 20 or polysorbate 80) [14]. For example, when comparing two of the highest‐grossing subcutaneous monoclonal drugs, Dupixent (dupilumab) is formulated with histidine, arginine, sucrose, and polysorbate 80 in a sodium acetate buffer [15] while Skyrizi (risankizumab‐rzaa) is formulated with trehalose, polysorbate 20, and sodium acetate [16].

However, combinations of excipients are reported to have unintuitive, non‐linear, and interactive effects [17, 18, 19, 20]. This complicates the ability to map the underlying property‐function relationships, intuitively predict performance, and efficiently select formulations via a rational design‐based approach. This is further complicated by the need for formulations to simultaneously satisfy multiple design criteria (e.g., thermal and colloidal stability, low viscosity, etc.), where excipients beneficial to one property might degrade performance of another. For example, high antibody charge density (influenced by formulation pH) can both improve colloidal stability (by increasing repulsive forces between individual macromolecules) and reduce thermal stability (inducing unfolding due to increased intramolecular repulsions) [18]. This, in turn, leads to cross‐pressures for individual formulation decisions driven by competing objectives. Therefore, successful antibody formulations must include combinations of excipients that balance these trade‐offs between metrics to have acceptable overall performance.

Furthermore, differences in physiochemical properties between individual antibodies limits the ability to extrapolate formulation performance from one to another [21, 22, 23]. This lack of a “one‐size‐fits‐all” approach is observed in the diverse array formulations utilized across the current landscape of approved antibody‐based therapies [7]. As such, formulations often need to be tailored for that specific therapy. This becomes even more acute for complex antibody‐based derivative biologics such as bi‐specifics and antibody‐drug conjugates, which can present novel stability challenges or introduce new formulation requirements (e.g., drug linker stability) [24]. As such, *de novo* formulation development for a novel antibody has historically relied on human experience and training, rational design, and high‐throughput screening, which can necessitate time‐ and material‐intensive formulation campaigns [25].

In contrast, recent efforts have demonstrated the potential for machine learning (ML) to effectively navigate these types of complex, multidimensional design spaces and map the underlying property‐function relationships [26, 27]. These are often paired with techniques such as Bayesian optimization (BO) where a surrogate model representing these relationships is used to suggest designs for subsequent experimentation [28]. Significant success has been seen in the fields of medicinal chemistry [29, 30, 31], pharmaceutics [32, 33, 34], and biotechnology [35, 36, 37, 38], where BO has been applied to guide the development of therapies. In parallel, automation platforms have become increasingly important engines for drug discovery [39, 40] and material design [41, 42], drastically increasing experimental throughput while also improving accuracy and precision. Previously, we successfully applied these tools for the development of novel polymer excipients for protein and drug stabilization [43, 44, 45]. Likewise, the pairing of these technologies is well‐suited to accelerate the process of formulation discovery.

Arosio and coworkers previously reported the feasibility of applying BO for formulating biologic drugs, utilizing ML to simultaneously optimize formulation features [46, 47]. Herein, we demonstrate the potential of this approach to be further accelerated with automation, while extending the scope of the multi‐objective optimization problem to alterative high‐dimensional objective spaces that explicitly evaluate features relevant for development of subcutaneously‐injectable formulations. This, in turn, establishes the scalability of this optimization strategy to significantly more complex formulation challenges faced in clinical product development. Toward this end, we developed an automation‐assisted formulation discovery pipeline driven by BO. Liquid handling robots were used for on‐demand, low‐volume, high‐throughput formulation in 96 well plate formats. This was paired with active learning to intelligently guide experimentation and testing toward high‐value formulations within the vast design space.

Through multiple rounds of active learning, we evaluated the ability of this system to simultaneously optimize parameters associated with thermal and colloidal stability, alongside high concentration viscosity [13, 48, 49]. Specifically, thermal stability was assessed through protein melting temperature [46, 50, 51], while colloidal stability was assessed through antibody diffusivity [47, 52, 53], which reflects the strength of intermolecular protein‐protein interactions in solution; at a given antibody concentration, formulations with stronger net‐repulsive interactions exhibit higher diffusivity while formulations prone to self‐association report suppressed values. Collectively, this demonstrates the potential of this strategy to leverage modest, experimentally feasible datasets to efficiently map a complex formulation design space and navigate to optimized designs.

## 2 Results and Discussion

### 2.1 Data‐Driven Discovery Pipeline for Antibody Formulation

To evaluate a ML‐driven approach for antibody formulation, we iterated formulation designs leveraging a Design‐Build‐Test‐Learn (DBTL) [54] cycle (Figure 1) to optimize a model bovine immunoglobulin G (bIgG) antibody across three objectives: maximization of diffusivity, maximization of thermal stability (defined by the melting temperature, *T_m_*), and minimization of viscosity. These objectives were chosen in‐line with needs for antibodies which must remain colloidally and thermally stable while retaining low viscosity when highly concentrated. Each cycle consisted of four primary steps: (1) automation‐assisted formulation and sample preparation; (2) performing biophysical characterization measurements; (3) training ML models to predict *T_m_*, diffusivity, and viscosity directly from formulation features; (4) proposing 24 new formulations to optimize target objectives using active ML. These newly proposed candidates were then formulated, characterized, and provided as additional training data to update models and begin the cycle again. Our approach here utilized two complete cycles to demonstrate the feasibility of improving each objective.

**FIGURE 1:**
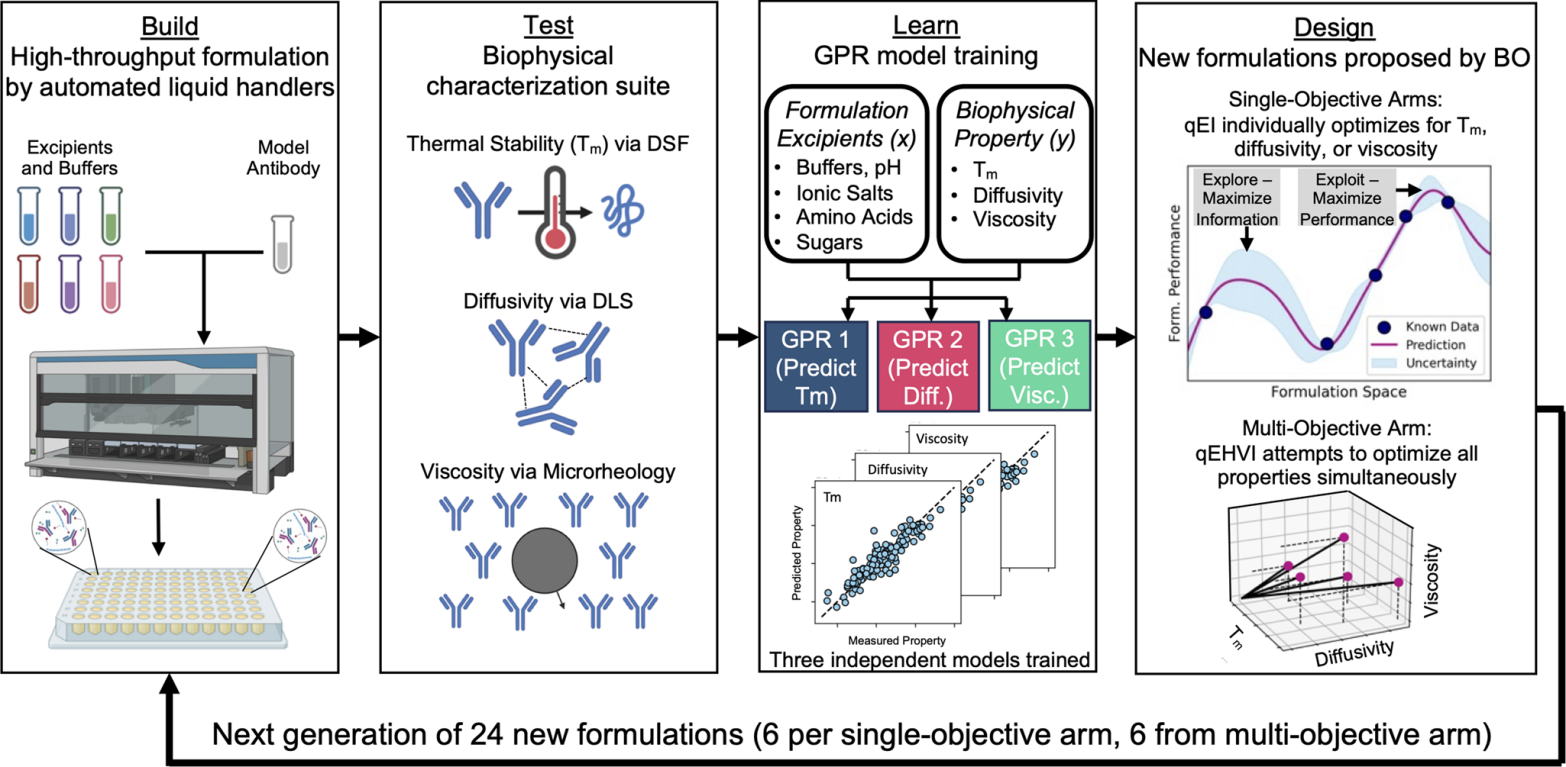
Overview of study. Build) Automated‐assisted formulation of bIgG by a Hamilton MLSTARlet liquid handling robot in 96‐well plates. Test) Biophysical characterization of bIgG formulations is performed to acquire thermal stability (T_m_) by differential scanning fluorimetry (DSF), diffusivity by dynamic light scattering (DLS), and viscosity data by high‐throughput microrheology. Learn) After initialization with seed data, objective‐specific Gaussian process regressors (GPRs) surrogate models are trained to predict T_m_, diffusivity, and viscosity of a given formulation. Design) Models are paired with expected improvement acquisition functions to optimize formulations for single and multiple objectives. Created in BioRender. Gormley, A. (2026) https://BioRender.com/72ot4pd.

#### 2.1.1 Initial Seed Library & Formulation Space

To generate initial data for our DBTL cycles, we performed an initial sampling of our formulation space. Clinical antibody formulations typically draw from a diverse combination of excipients to alter intermolecular interactions and enhance biophysical properties of formulations [7]. We attempted to capture this complexity within a streamlined formulation design space of GRAS excipients (Table S1). Each formulation consists of a combination of buffer (defined by buffering system and pH), L‐arginine, sucrose, and NaCl, each at an independently variable concentration within the specified range. Ranges for each excipient were selected to ensure typical concentrations used for clinical formations were accessible within the boundaries. Even with this streamlined design space, this yielded ∼800 000 000 unique formulations within a high‐dimensional design space when limiting continuous parameters to a step size of ±1% of their respective range. Exploring this design space at high resolution is experimentally intractable, necessitating selection of a subset of formulations that can cover the space and provide downstream ML models with a strong foundational information dataset to guide subsequent active learning [55]. Toward this end, 24 formulations were selected by Latin hypercube sampling (LHS) [56], a design of experiments (DOE) approach that efficiently samples the six‐dimensional space, with an individual “formulation” defined as a unique combination of excipient identities and concentrations (i.e., buffer system, pH, arginine, sucrose, and NaCl). This seed library provided a modest, synthetically feasible number of formulations that nevertheless provided a diverse array of excipient combinations.

#### 2.1.2 Build

The proposed 24 formulations were then prepared via automation assisted on‐demand formulation using a Hamilton MLSTARlet liquid handing robot. Each of the 24 formulations was prepared at multiple antibody concentrations (2.5, 5, 10, and 15 mg/mL for *T_m_* and diffusivity studies and 72, 90, and 120 mg/mL for viscosity studies) for downstream characterization. Importantly, this approach facilitated accurate, reproducible, combinatorial excipient mixing in a high‐throughput 96 well plate format. This provided sufficient experimental material for downstream characterization while simultaneously minimizing antibody consumption (approximately 10 mg of antibody for *T_m_* and diffusivity studies and 12 mg for viscosity study per formulation replicate).

#### 2.1.3 Test

After automation‐assisted preparation in 96‐well plates, formulation *T_m_*, diffusivity, and viscosity were then collected through a suite of high‐throughput characterization techniques. Differential scanning fluorometry (DSF) was used to collect information on thermal stability of the formulation by quantifying its *T_m_* at formulation concentrations. In parallel, dynamic light scattering (DLS) was used to determine diffusivity of the antibody in solution to obtain insight into changes in intermolecular interactions and colloidal stability. Finally, DLS was also utilized to capture viscosity at high antibody concentrations (up to 120 mg/mL) utilizing microrheology techniques. Experimental details of all techniques are described in Section 4.

#### 2.1.4 Learn

To efficiently predict the diffusivity, thermal stability, and viscosity of new formulations, we trained three independent Gaussian process regressors (GPRs) [57] directly from formulation feature vectors (see Section 4). Models provided forward‐looking predictions (*µ*) and uncertainty estimates (*σ*) of formulation behavior on any combinations of excipients and buffers in our streamlined design space.

#### 2.1.5 Design

Trained GPRs were then used to launch four independent BO campaigns, each targeting a different objective for formulation improvement. Three of the campaigns aimed to optimize a single biophysical parameter without regard for the other two (i.e., *T_m_* only). In contrast, the fourth arm attempted to consider all three parameters in order to select balanced formulations across multiple objectives. For each objective, six novel formulations that had not previously been evaluated were proposed by maximizing single objective or multi‐objective expected improvement (EI) acquisition functions (see Section 4). This batch of formulations was suggested by the function to facilitate two goals: (1) leveraging the model's understanding to select highly‐performant formulations, and (2) evaluating areas of the design space where model uncertainty is elevated, thus providing additional data to improve the model's understanding.

### 2.2 ML Models Guided Selection of Improved Formulations via Bayesian Optimization

The 24 seed formulations proposed by LHS successfully produced formulations with a wide range of melt temperatures, diffusivities, and viscosities across our set of formulation parameters. Across all four optimization arms, BO utilizing surrogate GPR models yielded new generations of candidates whose target properties significantly improved on the seed library (Figure 2A). With only two rounds of active learning, the diffusion campaign increased the mean diffusivity by 22.2%, the viscosity campaign lowered mean viscosity by 13.7%, and *T_m_* campaign raised mean melt temperature by 1.4^°^C, each change reaching statistical significance (p < 0.005). The expected‐improvement acquisition functions utilized in our approach (qEI [58] for single objectives, qEHVI for HV) explicitly balance exploitation of high performance regions with exploration of high uncertainty region; the broad inter‐quartile ranges of the dashed‐box “predicted” distributions in Figure 2A confirm that both facets were represented in every 6‐formulation batch. This explore‐exploit balance is built into the structure of the acquisition function rather than controlled by an explicit tunable parameter (see Section 4). Importantly, utilizing this approach, each arm was also able to identify individual high‐performing formulations with properties that exceed those in the seed library. Specifically, lead candidates from the *T_m_*‐, diffusivity‐, and viscosity‐optimizing arms were able to improve their target objective by 0.75%, 4.64%, and 7.67%, respectively, over the best‐performing seed formulation.

**FIGURE 2:**
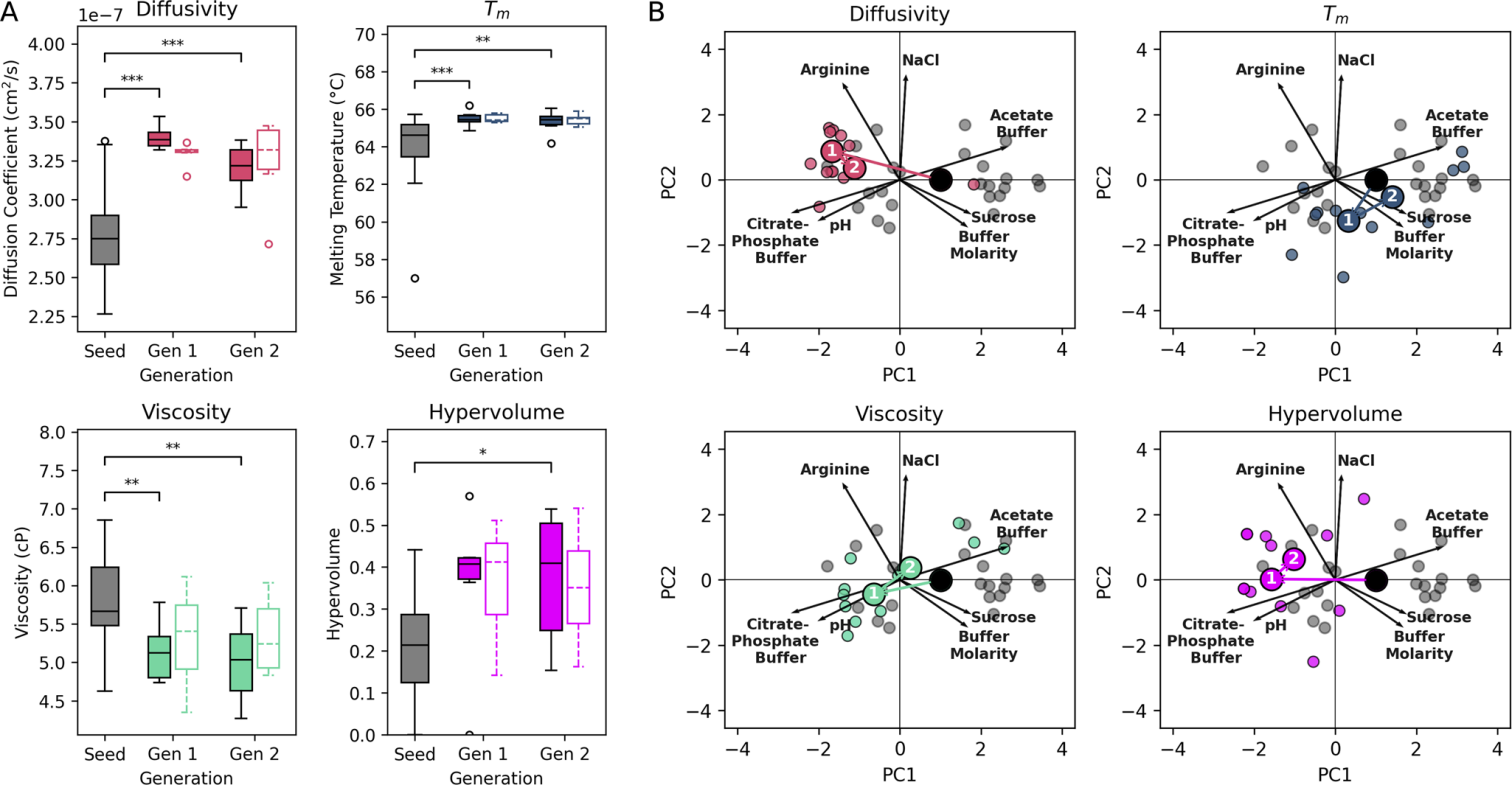
ML guides the design of bIgG formulation based on optimization objective. (A) Measurements (solid plots) and predictions (dashed plots) acquired for formulation diffusivity, T_m_, viscosity, and hypervolume after a single round (Gen 1) and two rounds (Gen 2) of active learning loops compared to seed formulations. Formulation predictions (µ) were determined from the GPR used by BO to propose that batch. Statistical significance was assessed between measured values for seed formulations and ML proposed formulations and determined by single tailed t‐test. ^*^(*p* < 0.05), ^**^(*p* < 0.005), ^***^(*p* < 0.0005), unlabeled pairs are not significantly different. (B) Principal component analysis (PCA) of ML‐guided bIgG formulations based on optimization objective demonstrating unique pathing through formulation space. Large numbered points are calculated centroids of proposed samples (colored points) from Gen 1 and Gen 2 formulations for each objective. All instances originate from the centroid of the Seed formulations (grey points).

The multi‐objective optimization (MOO) arm, steered by the q‐expected hyper‐volume improvement (qEHVI) [58], registered the largest gain; a 73.3% gain in mean normalized hypervolume (HV) was observed, illustrating that the joint surrogate captured the Pareto structure of the problem from the initial 24 formulations in the seed dataset. Furthermore, a lead candidate was identified that improved the metric by 21.9% over the best seed formulation. As HV represents the product of the three scaled objectives, the qEHVI function attempts to balance performance between them. To achieve this, the MOO explored multiple optimization strategies, selecting mutually non‐dominated formulations along the Pareto front. In practice, this led to diverse formulation designs that prioritized one or two properties while attempting to limit performance losses in the remainder. These strategies yielded varying levels of success. When biophysical properties of the MOO formulations were characterized and used to calculate associated HVs, the batch displayed inconsistent performance, leading to a wide range of HVs across the candidates. Interestingly, deeper inspection reveals that the composite metric preferentially favored the diffusivity/viscosity axis at the expense of thermal stability. This bias emerges as formulations optimized for diffusivity and viscosity demonstrated strong cross‐over performance in both objectives (Figure S1). qEHVI therefore rewarded candidate formulations that drove the dual colloidal objectives aggressively while maintaining moderate thermal stability, yielding HV improvements. This result suggests the need to articulate explicit objective floors in future multi‐objective campaigns.

Interestingly, while the first generation of BO candidates yielded significant improvement over the seed library, minimal further improvement was observed after the second round of BO. For diffusion and viscosity, Gen 2 medians were statistically indistinguishable from Gen 1, and no Gen 2 formulation exceeded the best Gen 1 candidate in any objective besides viscosity (Figure 2A). In silico projections of a hypothetical Gen 3 likewise failed to predict further improvement (Figure S2) even when using alternative acquisition functions that favored exploitation, such as the q‐upper confidence bound (qUCB, β = 0.1) [59] and a pure‐exploitation posterior‐mean (“Greedy mean”) batched via the constant‐liar scheme [60] (Figure S3), indicating that the exploitable region of design space was largely exhausted.

This rapid saturation is characteristic of BO in smoothly varying physicochemical spaces: once qEI has exploited the principal gradient, remaining unexplored niches confer diminishing marginal utility. This observation suggests that one or two DBTL rounds may suffice to reach near‐optimal performance, greatly reducing experimental burden relative to classical factorial and high‐throughput screens.

To better understand how the model optimized each of these target features with respect to the underlying formulation parameters, principal component analysis (PCA) was used to visualize the locations of formulations within the design space (Figure 2B). Centroids were calculated for each generation to understand pathing through the design space by BO for each of the four respective optimization goals. Interestingly, each arm of the BO led to proposed formulations centered in distinct formulation regions. For example, optimization of diffusivity yielded formulations largely in Quadrant II of design space, which represent formulations with high arginine, low sucrose, and low osmolarity (Figure S4). In contrast, the *T_m_* arm proposed formulations in Quadrant IV, representing low arginine and high sucrose. The fact that diffusivity‐optimized and *T_m_*‐optimized centroids identified by BO occupy opposite regions of the design space demonstrates the inherent tradeoff when optimizing both parameters simultaneously via MOO. The multi‐objective optimization arm yielded formulations with the largest spread throughout the design space, with individual candidates in each of the four quadrants. This likely arises from the multiple strategies explored by the MOO to balance the three competing objectives, as well as the higher uncertainty inherent in the HV parameter. However, the majority of the MOO candidates are in Quadrant II and III, which reflects the dominant strategy of favoring candidates with better diffusivity and viscosity. Analysis of the individual formulations proposed by each arm demonstrates the propensity of the BO to drive excipient compositions toward the extremes of the design space (Figure S4), indicating that further improvement of formulation properties might be achieved with an expanded design space.

### 2.3 Exploring Property‐Function Relationships of Individual Formulation Components

As objective‐specific BO was generally successful in identifying generation‐on‐generation improvements in formulation behavior, we sought to understand the formulation designs that underlie objective‐specific optimizations. Explainable artificial intelligence (exAI) techniques are essential for building trust and ensuring that models make predictions based on sound formulation principles rather than spurious correlations [61]. Using SHAP (Shapley Additive Explanations) [62, 63], we probed the objective‐specific GPR models for *T_m_*, diffusivity, and viscosity to validate their reasoning and extract formulation insights. Here, positive SHAP values indicate positive contributions to *T_m_*, diffusivity, and viscosity, while negative SHAP values suggest negative contributions, and we use the mean absolute SHAP value of a feature as a proxy for its overall importance to model predictions. In order to evaluate these trends over the entire dataset, SHAP values were computed for all 72 formulations across all three single objective models (Figure 3A–C). This summary analysis reveals key drivers of formulation performance for each objective. Comparing SHAP summary plots across *T_m_*, diffusivity, and viscosity we observe trends that are largely in line with reported literature [19, 64]. Diffusivity is impacted most by arginine and charge state of the antibody (which is largely controlled by buffer parameters). Arginine is well known to reduce attractive interactions in protein formulations by transiently interacting with hydrophobic regions on the protein and increasing net repulsive forces, reducing self‐association and improving diffusivity [65]. While the “ideal” buffer pH will vary significantly from antibody to antibody based on the isoelectric point (pI), the relatively acidic pI of the model bIgG antibody used herein leads to improved diffusivity with increasing pH, as this will increase the net repulsive forces between individual antibodies in solution. Interestingly, a negative correlation is observed between buffer molarity and diffusivity, which is likely due to the fact that buffering salts may also produce charge‐shielding effects, reducing the strength of intermolecular repulsive forces [66]. Additionally, we observe that SHAP values for viscosity are dominated by antibody concentration and arginine content. As with diffusivity, arginine's ability to reduce intermolecular interactions—particularly hydrophobic interactions that increasingly dominate at higher protein concentrations where macromolecules are forced into closer proximity—contributes to its ability to significantly reduce viscosity of formulated antibody [65]. Interestingly, *T_m_* SHAP analysis suggests concentration, arginine, and pH as the most impactful features. While classically, sucrose might be anticipated to be the largest driver of mAb thermal stability due to its ability to modify the protein hydration layer [64], SHAP analysis reveals that the model bIgG is likely highly charge‐sensitive requiring careful balance of pH and ionic shielding. This protein‐specific identification of dominant formulation features is a primary benefit of utilizing explainable AI approaches.

**FIGURE 3:**
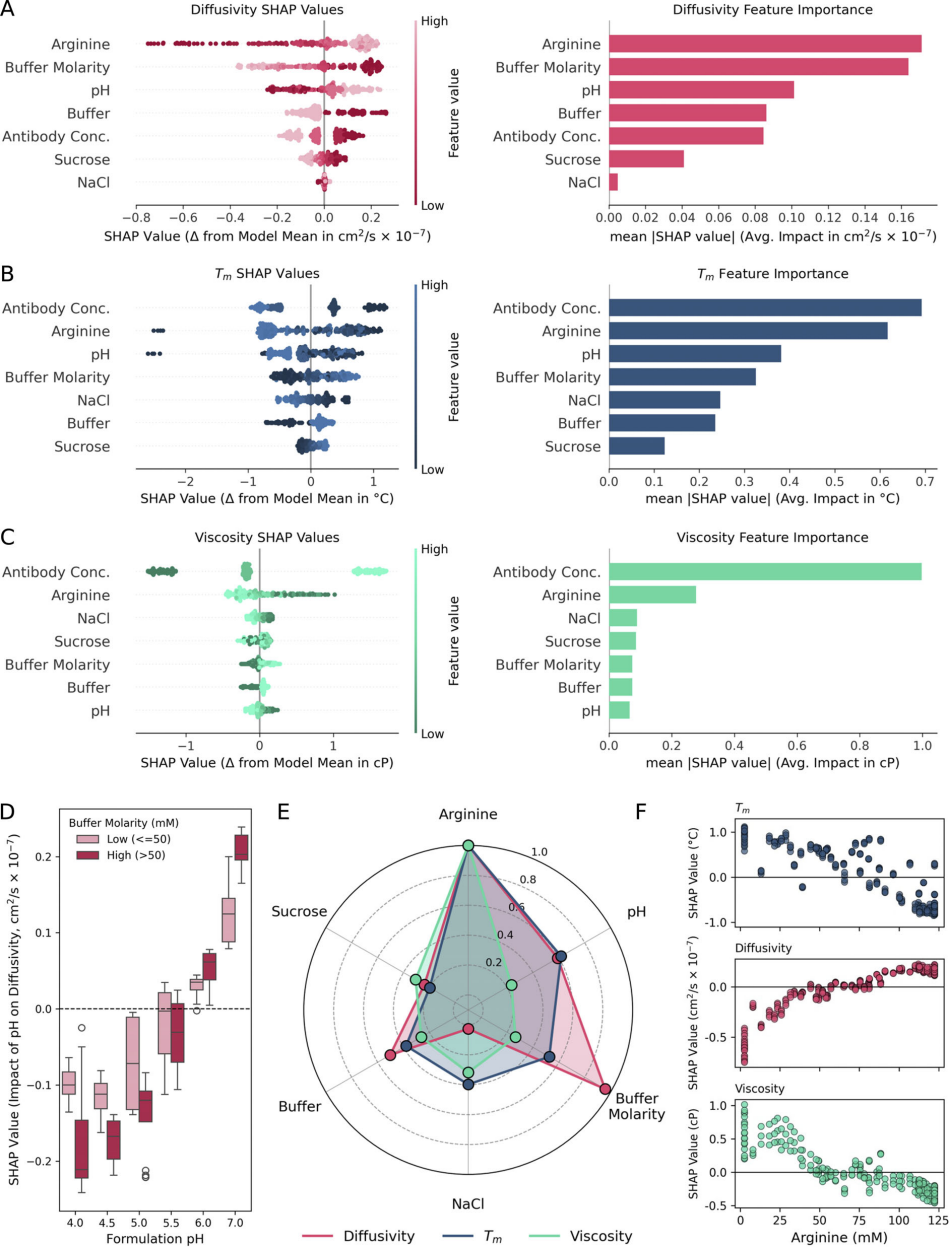
ExAI analysis reveals distinct priorities in formulation composition for each objective. (A–C) Summary of SHAP values for GPR models calculated from available data after all rounds of active ML cycles. (Left) Each point corresponds to a uniquely evaluated formulation, and the point's position along the *X*‐axis shows the impact of a feature on predicted diffusivity (A), T_m_ (B), and viscosity (C) respectively. (Right) SHAP derived feature importance values across all model features for diffusivity, T_m_, and viscosity respectively. SHAP derived feature importance are taken as the mean absolute SHAP value (|SHAP value|) to demonstrate the average impact of a feature on model predictions for each target. (D) Comparison of diffusivity SHAP values for formulation pH in low and high buffer molarity regimes. (E) Normalized mean absolute SHAP values calculated for diffusivity, T_m_, and viscosity models to compare relative feature importance across the three models. (F) SHAP dependence plots for arginine content, demonstrating impact of the feature on T_m_, Diffusivity, and viscosity model outputs across the full range of arginine concentrations evaluated in the study.

Furthermore, through SHAP analysis, nuanced biophysical phenomena can be observed. While diffusivity improves with increasing pH due to the antibody's low pI, this effect is enhanced with increasing buffer molarity (Figure 3D). This is likely due to the fact that the antibody itself as well as other formulation components (i.e., arginine) can also influence overall pH of the solution. As such, increasing molarity of the core buffering agent allows it to dominate. In contrast, low pH buffers see a negative influence of buffer molarity on diffusivity. This is likely due to the increasingly dominating effect of the buffer driving the pH of the system closer to the pI of the antibody [19], as well as the increased charge shielding effects inherent with higher salt content [66], both of which is collectively increase potential for attractive intermolecular interactions and decrease diffusivity.

When these mean absolute SHAP values are scaled relative to arginine (Figure 3E), its overarching dominance across all three objectives is readily apparent. While the diffusion model assigns a similar weight to buffer molarity (∼0.95), arginine far surpasses all other features like pH (∼0.6) and excipients such as sucrose (∼0.2) or NaCl (<0.1). Weights are even more lopsided in favor of arginine for the *T_m_* and viscosity models. This positions arginine as a key excipient in our formulation space and with regard to our model bIgG antibody. However, arginine demonstrates both synergistic and antagonistic trade‐offs depending on a given optimization objective (Figure 3F). For example, increasing arginine concentration correlates with increasingly positive SHAP values for diffusivity (middle panel) and increasingly negative SHAP values for viscosity (lower panel), promoting favorable diffusivity and rheological properties simultaneously. However, this comes at the expense of *T_m_* depression (upper panel), where similar increases in arginine concentration are predicted to decrease the thermal stability of bIgG significantly. These findings by SHAP largely explain the results of our two DBTL cycles in which formulations that were optimized for diffusivity or viscosity generally performed well in both objectives, but often demonstrated low thermal stability (Figure S1). In contrast, formulations that were optimized for *T_m_* generally demonstrated lower diffusivity and higher viscosities. In the multi‐objective HV arm of the study, most designs intended to maximize our qEHVI acquisition function localized to an area of PCA space similar to the diffusion‐ and viscosity‐optimized formulations (Figure 2B). This was a synergistic design space where high performance for two objectives could be simultaneously obtained. In contrast, the distant high‐*T_m_* space satisfied high‐performance for only a single objective, leading to the MOO arm clearly biasing formulation selection away from this area.

Beyond identifying influential features, we further interrogated the underlying response surfaces to characterize the nature of these feature contributions. Partial dependence analysis across the design space (Figure S5) revealed both monotonic responses and clear non‐linearities for several features, with local optima visible in the arginine, pH, and sucrose slices. Fitting an additive linear surrogate to each trained GPR (Table S2) quantified the relative contributions of main effects and interactions: the diffusivity and viscosity models were largely additive while *T_m_* was poorly captured by an additive approximation, indicating a substantial role for interactive effects. Friedman's H‐statistic [67] (Figure S6) localized these interactions to the coupling between arginine concentration and the buffer conditions (both pH and molarity) in the *T_m_* model. Collectively, these analyses establish that thermal stability is shaped by non‐additive coupling between excipients to a greater extent than the other formulation properties.

### 2.4 Post‐Hoc Analysis of Model Learning

After two DBTL cycles, each GPR model trained on the full set of 72 formulations achieved high predictive fidelity. Group k‐fold cross‐validation (10 folds, with all replicates of each formulation in the same fold) was used as the primary metric for model performance evaluation (Figure S7). Under this scheme, the viscosity model demonstrated the highest accuracy of R^2^ = 0.87, while the diffusion and *T_m_* models demonstrated R^2^ values of 0.80 and 0.70, respectively. Predictive performance was also broadly maintained regardless of formulation novelty relative to the training set (Figure S8). To assess whether the GPR architecture was necessary to achieve this level of performance, we benchmarked the trained models against a panel of simpler regression baselines, including linear, polynomial, and tree‐based models (Figure S9). For the diffusion coefficient and viscosity objectives—whose response surfaces were largely additive in nature (Section 2.3)—several simpler architectures achieved comparable predictive accuracy. However, none matched GPR performance on *T_m_* (R^2^ < 0.65 for all alternatives), where non‐additive coupling between excipients dominates the response surface. This positions the GPR model as the overall top performer across the three objectives, which was essential for an effective multi‐objective optimization campaign.

To complement this overall performance assessment, the learning rate of the three GPR models—their ability to translate additional data into improved model performance—was assessed using a modified leave‐one‐out cross‐validation approach (LOOCV) that mimicked the sequential expansion of the active learning campaign (Figure 4A). The “left out” formulation was predicted using a model progressively trained on more data—from the initial seed library (24 formulations) to the entire library minus the held‐out formulation (71 formulations). By repeating this process and holding out a different formulation from Generation 1 or 2 each time, the entire non‐seed library (48 formulations) was predicted and aggregated to compute overall R^2^ and mean absolute error (MAE) for each model as a function of training set size.

**FIGURE 4:**
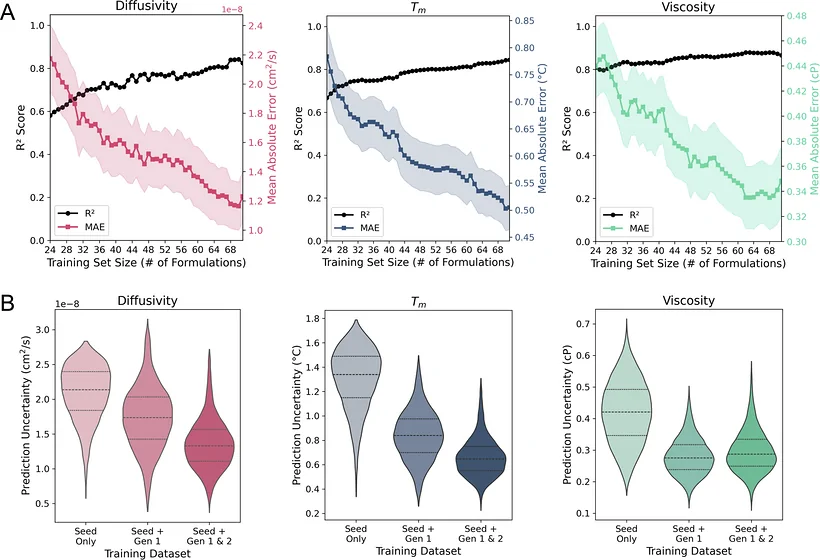
ML models demonstrate high accuracy and prediction confidence in low data regimes. (A) Learning rate analysis for the three models, tracking improvement in predictive accuracy in response to additional training data. MAE mean is presented with standard error of values across the 48 arms of LOOCV. (B) Gen‐on‐gen improvement in model uncertainty (standard deviation of posterior probability density function) when predicting over the entire design space (represented by a survey of 10 000 formulations in silico).

Impressively, even using solely the seed library for training, the *T_m_* and diffusivity models demonstrated reasonable R^2^ values of 0.68 and 0.59, respectively, while the viscosity model demonstrated a strong R^2^ of 0.80. The latter is likely due to the uniquely dominating influence of antibody concentration on viscosity *vis‐à‐vis* other formulation features (Figure 3C), enabling the model to make reasonably accurate predictions without needing to fully understand the nuances of the formulation itself. As the models receive additional formulation data in their training set, these performance metrics consistently increase. When given the full training set, R^2^ scores for all models exceed 0.82. Alongside improvements in R^2^ scores, error is significantly reduced for all GPRs as more data is provided to the model, with 35.6%, 43.4%, and 20.8% reductions in MAE for the *T_m_*, diffusivity, and viscosity models, respectively. Notably, the final MAE for *T_m_* (0.50 °C) and viscosity (0.35 cP) is comparable to the average experimental error (0.48 °C and 0.34 cP, respectively), suggesting the models are approaching the noise floor of the measurements. In contrast, the diffusivity MAE (1.23 × 10^−^^8^ cm^2^/s) remains higher than the experimental error (6.83 × 10^−^^9^ cm^2^/s), likely reflecting the exceptionally low relative noise of that technique. Overall model error reflects both aleatoric (intrinsic) and epistemic (model‐related) uncertainty [68]. As such, convergence of model MAE to the experimental noise indicates that the models have largely captured the learnable patterns in the data, with residual error dominated by irreducible measurement noise.

Ultimately, the value of these models lies in their ability to make accurate and confident predictions for novel formulations across the entire design space. To assess this, we quantified the generation‐on‐generation reduction in global model uncertainty by predicting the properties of 10 000 formulations in silico (Figure 4B). The analysis revealed a significant decrease in the mean predictive uncertainty (the standard deviation of the GPR posterior) for all three objectives: 49.1% for *T_m_*, 34.9% for diffusivity, and 29.5% for viscosity over the course of the campaign (Table S3). This global reduction demonstrates that the active learning strategy, by balancing exploration of high‐uncertainty regions with exploitation of high‐performance regions, successfully builds a more confident and generalizable model of the entire formulation landscape. As the model receives more data, its probability density function (PDF) shifts. Ideally, predictions for novel formulations outside of the training data should not only become more accurate, but also more confident as the model develops a more precise understanding of the relationship between the design features and target objective values (Figure S10). Further, this improvement is not merely localized to the sampled points but is propagated across the design space via the GPR's covariance kernel, validating the model's utility for future in silico screening and optimization. The observed plateau in uncertainty reduction for the viscosity model after the first generation is consistent with its rapid convergence to the noise floor, indicating that it reached a state of diminishing returns on additional data sooner than the other models.

## 3 Conclusion

Efficient optimization of antibody formulations remains a critical bottleneck in biologics development. Next‐generation antibody therapies often require concentrations ≥100 mg/mL for subcutaneous delivery, but at such concentrations antibodies can exhibit extreme viscosities and stability issues, creating formidable hurdles in development, manufacturing, and administration. Conventional formulation campaigns rely on trial‐and‐error excipient screening to mitigate aggregation and viscosity, yet the complexity of protein‐excipient interactions makes this process time‐consuming and inefficient. Identifying a stable, low‐viscosity formulation can demand extensive experimentation and material, slowing the path to the clinic and straining production resources. These challenges underscore the need for more data‐driven, accelerated formulation design strategies.

In this work, we demonstrate a ML‐ and automation‐assisted pipeline to streamline antibody formulation development. The platform implements a Design‐Build‐Test‐Learn framework, integrating robotics‐assisted formulation preparation, high‐throughput biophysical characterization, and GPR surrogate models to navigate the antibody formulation design space. Over two iterative DBTL rounds guided by BO, the system was able to successfully target key formulation objectives: raising thermal unfolding temperature, increasing protein diffusivity, and lowering viscosity. Additionally, the multi‐objective optimization acquisition function used in this approach demonstrated the ability to optimize across these parameters simultaneously, intelligently negotiating between trade‐offs to maximize overall performance. Remarkably, robust surrogate models trained on only 72 formulations (which used less than 6 g of antibody in total) captured the underlying property‐function relationships. This outcome highlights the power of active learning to rapidly converge on optimal formulations from modest datasets, echoing recent evidence that ML‐guided experimentation can dramatically improve data efficiency in pharmaceutical development. Beyond performance gains, a pivotal advance of this study lies in moving beyond prediction to mechanistic understanding through the application of explainable AI. By leveraging SHAP, we deconstructed the GPR model to assign a quantitative contribution of each excipient to each training feature, thereby elucidating the distinct synergistic and antagonistic roles of specific formulation components. These mechanistic insights validate known biophysical trends (for example, arginine‐mediated suppression of self‐association) and illuminate the complex interplay of factors in a multi‐objective formulation. Such explainable models move beyond black‐box predictions, providing a rational foundation to guide excipient choices and balance trade‐offs in antibody formulation.

This work also builds upon a rapidly growing body of research demonstrating the power of machine learning, and Bayesian optimization in particular, to accelerate biologic formulation development—from the optimization of thermal and interfacial stability for antibody fragments [46] and the multi‐objective formulation of monoclonal antibodies [47], to the computational prediction of antibody‐excipient interactions [69] and the development of formulations in adjacent biologic modalities such as vaccines [70]. The present study extends this foundation by bringing together several complementary capabilities within a single, integrated pipeline. By incorporating high‐concentration viscosity as an explicit optimization objective alongside thermal and colloidal stability, we directly target the property that most directly governs syringeability at the concentrations required for subcutaneous delivery, complementing efforts that have navigated colloidal behavior through dilute‐solution descriptors [47]. While prior formulation development efforts have sought to reduce high‐concentration viscosity through empirical excipient screening [71], the present framework incorporates viscosity directly as an optimization target within the closed‐loop active learning campaign. By integrating automated, on‐demand formulation preparation into this prospective, closed‐loop DBTL cycle, we further streamline the experimental workflow and lay the groundwork for increasingly autonomous formulation campaigns. In addition, by applying explainable AI to interrogate the learned response surfaces, we extend the role of the surrogate model beyond candidate proposal to the mapping of the property‐function relationships that govern formulation behavior. Our approach is also highly complementary to the expanding body of work that applies machine learning to predict the developability of antibody panels from sequence‐, structure‐, or surface‐derived features [22, 72, 73]. Where those efforts identify which antibodies are most developable within a common formulation context, the present framework instead tailors a formulation to a given antibody. These represent complementary halves of the same overarching challenge, and their eventual integration—using molecular features to anticipate developability and inform formulation optimization models via transfer learning—offers a particularly promising direction for the field.

In practice, the selection of a lead formulation from a multi‐objective optimization campaign depends on the intended product profile and the relative weight assigned to each property. For example, a subcutaneously administered high‐concentration antibody places a premium on injectability, making low viscosity at high concentration an essential property, while an intravenously administered formulation at lower concentration may tolerate higher viscosity in exchange for improved thermal stability and shelf life. A key advantage of the surrogate‐based framework developed here is its flexibility in supporting these context‐specific decisions: once trained, the GPR models enable user‐defined constraints to be applied to identify formulations satisfying any given combination of design requirements. This positions the pipeline not as a tool that returns a single “optimal” formulation, but as a framework that maps the formulation design space such that any downstream selection criterion—whether dictated by route of administration, dosing regimen, storage requirements, or regulatory considerations—can be applied to identify suitable lead candidate(s) to carry forward for downstream development.

In establishing this framework, we focused on a single model antibody, bovine IgG, which allowed us to demonstrate the full Design‐Build‐Test‐Learn pipeline end‐to‐end. Of course, as in any formulation campaign, the quantitative response surfaces learned here reflect the physicochemical characteristics of this particular molecule. However, several aspects of these findings may extend beyond bIgG: certain effects, such as the viscosity‐reducing potential of arginine, are well‐documented across an array of antibodies and may represent broadly transferable design rules, whereas molecule‐specific factors such as optimal pH are expected to vary with the macromolecule's isoelectric point and would likely require the incorporation of antibody‐level physicochemical descriptors into the surrogate models, offering a natural route to implementing transfer learning across candidates. Furthermore, because the automation toolkit, multi‐objective active learning pipeline, and *post hoc* interpretability analyses developed here are not inherently specific to bIgG, they could likewise be readily redeployed for new antibodies. Looking forward, there are several promising extensions of this approach. The pipeline could be expanded to broader excipient spaces and applied across multiple antibody candidates to derive generalizable formulation design rules. The framework could also be extended to the ultra‐high protein concentrations (>200 mg/mL) that represent the current frontier of bioformulation challenges and/or accommodate additional optimization objectives beyond those explored here, further accelerating the development of stable, low‐viscosity biologics.

Finally, this work represents a step toward the realization of fully autonomous self‐driving laboratories (SDLs) [54], which promise to execute the entire scientific method—from hypothesis generation to experimental validation and learning—with minimal human intervention. Such systems have the potential to not only dramatically compress drug development timelines and enhance research reproducibility but also unlock the capacity to rapidly engineer novel and more effective medicines to meet urgent global health needs.

## 4 Experimental Section/Methods

### 4.1 Materials

All reagents were purchased from Sigma–Aldrich unless specified otherwise. Bovine IgG (bIgG) was purchased from MP Biomedicals.

### 4.2 Automation‐Assisted on‐Demand Antibody Formulation

To facilitate automated and on‐demand formulation of bIgG, a Hamilton MLSTARlet liquid handler was utilized to perform formulation preparation and necessary dilutions. Stock solutions for each formulation component were prepared in deionized water and filtered using a 0.2 µm PES filter prior to use. For *T_m_* and diffusivity studies, stock solutions of bIgG were first prepared in deionized water at 30 mg/mL, triple‐filtered using 0.05 µm PS filters to remove aggregates, then diluted to 20 mg/mL. Reagent and bIgG stock solutions were then loaded into the Hamilton ML STARlet liquid handler to prepare formulated bIgG in 96 well plates. Custom Python software was used to convert formulations compositions into actionable transfer steps that were then implemented automatically. Each excipient was available at 2–3 stock concentrations to facilitate formulation with high precision and accuracy while simultaneously ensuring the entire formulation design space remained accessible via a legitimate series of transfer steps. For each combination of excipients to be evaluated, working formulation stocks were first prepared from excipient stocks at 3x of the final intended concentration. These 3x formulation stocks were then combined with the bIgG stocks and additional deionzied water as needed to produce formulated antibody solutions at the correct concentrations of both antibody and excipient. For viscosity studies, stock solutions of bIgG were prepared at 200 mg/mL. 200 nm polystryene beads were then added to the solution to yield a 180 mg/mL solution with 0.1 wt.% bead. This solution was then used to prepare formulated antibodies at high concentrations. Antibody was formulated at 2.5, 5, 10, and 15 mg/mL for *T_m_* and diffusivity characterization studies, and at 72, 90, and 120 mg/mL for viscosity characterization studies. At each concentration, antibody formulations were prepared in triplicate for *T_m_* and diffusivity measurements, and in quadruplicate for viscosity measurements. bIgG stock solutions for the entire study were prepared immediately prior to seed library preparation and analysis. These same stocks were used for preparation for each round of DBTL to eliminate batch‐to‐batch variability. Likewise, stocks of individual excipients were prepared once at the start of the study and used for all rounds.

### 4.3 High‐Throughput Characterization

#### 4.3.1 Thermal Stability via DSF

Thermal stability of the formulated antibody solutions was evaluated by DSF using a QuantStudio 3 (Thermo Fisher Scientific) real‐time PCR system and established techniques [74]. A Sypro Orange solution was first prepared in deionized water and filtered using a 0.45 µm hydrophilic PTFE filter prior to use. Samples were then prepared by addition of 45 µL of the formulated antibody solutions were transferred to a PCR plate, followed by the addition of 5 µL of the Sypro Orange solution. The plate was then loaded into the instrument and formulations were subjected to a heat ramp from 25°C to 95°C at a rate of 1°C/min. Fluorescence (λ_Ex_ = 520 ± 10, λ_Em_ = 558 ± 11) was measured over the course of the ramp. Automated analysis was used to calculate the first protein melting event by applying a Savgol filter to pre‐process data and then curve fitting the Boltzmann sigmoid equation (Equation 1). Melt temperature (*T_m_*) was defined as the inflection point of the curve fit. In cases where multiple thermal transitions were observed, likely corresponding to denaturation of different domains of the antibody, the first transition was used for *T_m_* calculation to provide a more conservative estimate of thermal stability. Each formulation was evaluated at four antibody concentrations (2.5, 5, 10, and 15 mg/mL), each with three experimental replicates prepared and measured.

(1) $$y\left(x\right)=A_{2}+\frac{A_{1}-A_{2}}{1+\exp\left(\left(x-x_{0}\right)/dx\right)}$$

#### 4.3.2 Diffusivity Measurements via DLS

DLS measurements [75] were performed on a DyanaPro Plate Reader III (Wyatt Technologies) and analyzed using the accompanying Dynamics 7.10 software package. 96‐well plates containing the formulated antibodies were centrifuged at 2000 g for 5 mins at room temperature to remove microbubbles prior to analysis. For each sample, eight repeat acquisitions with 8 s acquisition time were collected at 25°C. A data filter was applied to all data to assess data quality, following the Wyatt Technology default parameters: minimum amplitude = 0, maximum amplitude = 1, baseline limit = 1± 0.01, polydispersity ≤ 40%. Diffusion coefficient (*D_c_*) measurements for that formulation replicate were then calculated from the average of the cumulant autocorrelation function. Each formulation was evaluated at four antibody concentrations (2.5, 5, 10, and 15 mg/mL) with each three experimental replicates prepared and measured per concentration.

#### 4.3.3 Microrheology for Viscosity Measurements

Viscosity measurements of antibody formulations were performed by using a high‐throughput DLS‐based technique to track the diffusion of 200 nm diameter polystyrene beads added as tracer particles [48]. 384‐well plates containing the formulated antibodies along with 200 nm polystyrene microspheres were first centrifuged at 2000 g for 5 mins at room temperature to remove microbubbles prior to analysis. For each well, ten consecutive 20s acquisitions were collected at 25°C using optimized laser power and attenuation settings. A data filter was applied to all data to assess data quality, following the Wyatt Technology default parameters: minimum amplitude = 0, maximum amplitude = 1, baseline limit = 1 ± 0.01, polydispersity ≤ 40%. Regularization was used to obtain the apparent radius of the microsphere. Viscosity of the solution was then calculated using the Stokes‐Einstein equation (Equation 2) to relate measured radius (R*_h app_*) of the tracer bead to the known radius (R_h Bead_) and determine the shift in viscosity at fixed temperature (Equation 3). Viscosity was measured 24 h after initial formulation to avoid transient viscosity effects arising from macromolecular network rearrangement. Each formulation was evaluated at three antibody concentrations (72, 90, and 120 mg/mL), with each four experimental replicates prepared and measured per concentration.

(2) $$R_{h}=\frac{k_{B}T}{6\pi \eta D}\;$$

(3) $$\eta_{2}=\frac{\eta_{1}R_{h\;app}}{R_{h\;Bead}}$$

### 4.4 Machine‐Learning Surrogate Models

Antibody formulations were encoded as seven‐dimensional physicochemical vectors comprising buffer molarity, buffer identity, buffer pH, NaCl concentration, sucrose concentration, arginine concentration, and protein concentration. Continuous features entered the model unaltered, whereas the categorical buffer was label‐encoded (1  =  acetate, 2  =  citrate‐phosphate) with discrete values. Objective‐specific MinMaxScalers from Sci‐Kit Learn subsequently mapped each feature to [0, 1]; distinct scalers were retained for *T_m_*, *D_c_*, and viscosity (*η*) so that the scale of one objective could not influence another. Targets were scaled analogously and stored alongside formulation identifiers that were later used to enforce group‐wise splits in cross‐validation. For each objective the relationships between formulations and biophysical characterization measurements were modeled using GPR to capture nontrivial, nonlinear mapping and to support active learning as GPRs naturally provide both mean (*µ*) and uncertainty (*σ* ^2^) on predicted points. The GPR surrogate was instantiated with BoTorch's SingleTaskGP [58] in which covariance is modelled by an isotropic squared‐exponential kernel:

(4) $$k\;\left(\vec{x},\vec{x^{\prime }}\right)=\sigma^{2}\;\exp\left(-\frac{{\left|\left|\vec{x}-\vec{x^{\prime }}\right|\right|}^{2}}{2l^{2}}\right)+\sigma_{n}^{2}$$

where $\vec{x}$ is the feature vector of the formulation, and σ, *l*, σ_*n*_ are kernel hyperparameters. These parameters were optimized by maximizing the exact log‐marginal likelihood. Predictive performance was assessed with 10‐fold group k‐fold cross‐validation in which all repeats from the same ‘Formulation ID’ reside in the same fold; this choice ensures that performance is evaluated only on formulations not seen during training, preventing optimistic sampling bias that could arise from evaluating each formulation across multiple protein concentrations. Predictions across all folds were collected and model performance was then assessed via coefficient of determination (R^2^ value) and mean absolute error (MAE), calculated between predicted and measured values.

### 4.5 Candidate Formulation Generation

BO was employed to navigate the seven‐dimensional formulation domain and prioritize new experimental queries. Each property (T_m_, *D_c_*, or *η*)—was first optimized in isolation using its dedicated Gaussian‐process surrogate. All coordinates were expressed in objective‐specific [0, 1] space obtained by Min–Max scaling of the original experimental limits; the transformation is invertible, guaranteeing that proposed designs remain within chemically feasible bounds (i.e., do not exceed the limits defined in Table S1). A fixed batch size of q  =  6 for each objective was imposed to align with plate‐based synthesis throughput. During each round, single‐objective optimizers maximized the q‐exclusive expected improvement (qEI) acquisition function [58], defined as:

(5) $$qEI\;\left(X\right)=E\left[\max_{1\leq i\leq q}{\left(f_{i}-f^{+}\right)}_{+}\right]$$

where *f* ^+^ denotes the best observed response.

The expected‐improvement acquisition function does not contain an explicit tunable exploration/exploitation parameter (such as the β coefficient of upper‐confidence‐bound acquisitions). Instead, the exploration/exploitation balance was built into the expectation itself: the acquisition value at a candidate point depends on the full posterior distribution at that point, $N(\mu \left(x\right),\sigma^{2}\left(x\right))$ and is sensitive to both the posterior mean and the posterior variance. For a single‐point evaluation this decomposes analytically as

(6) $$\mathrm{EI}\;\left(x\right)=\left(\mu \left(x\right)-f^{+}\right)\Phi \left(z\right)+\sigma \left(x\right)\;\phi \left(z\right)$$

where

(7) $$z=\frac{\mu \left(x\right)-f^{+}}{\sigma \left(x\right)}\;$$

Φ and ϕ are the standard‐normal CDF and PDF, and *f* ^+^ is the best observed response.

The first term rewards candidates whose predicted mean exceeds the incumbent best (exploitation), while the second term rewards candidates with high posterior uncertainty regardless of their predicted mean (exploration).

qEI generalized this to batch evaluation via Monte Carlo sampling from the posterior, preserving the same property: posterior samples in the upper tail of an uncertain candidate's predictive distribution contribute to the expected improvement even when the candidate's mean is unremarkable. The balance therefore evolved implicitly as data accumulates across the campaign—regions that had been densely sampled have small σ and contribute primarily through the exploitation term, while unexplored regions retain large σ and remain attractive through the exploration term until they were sampled.

A quasi‐Monte Carlo (QMC) sampler [76] was used to approximate the expected improvement by drawing samples from the model's multivariate normal posterior distribution using Sobol sequences [77], improving accuracy of acquisition values. For viscosity, the training targets were stored as (1 – *η*) so that all objectives share a maximization convention; the same transformation was applied to *f* ^+^ when evaluating qEI. qEI was evaluated at 500 uniformly spaced Sobol seeds and the best local optimum across restarts was taken as the final batch. These six points were inverse‐scaled to experimental units and screened to ensure valid buffer/pH pairs are proposed before automation‐assisted formulation.

Upon completing the three single‐objective optimizations, *T_m_*, *D_c_*, and *η* GPR models were utilized jointly to perform multi‐objective optimization (MOO). For this, we maximized the q‐expected hypervolume improvement (qEHVI) acquisition function [58]:

(8) $$qEHVI\;\left(X\right)=E_{F∼p\left(F|D\right)}\;\left[\max!\left(H\left(P\cup F\right)-H\left(P\right),0\right)\right]$$

where *X* is the batch of q candidates, *F* is the posterior samples at those points, P denotes the current Pareto set, and *H*( · ) represents the dominated hypervolume with respect to a fixed reference point r  =  (0, 0, 0). qEHVI quantified the expected increase in dominated hyper‐volume of the Pareto set when a batch of size six was added, with *η* again transformed to (1 – *η*) so that all objectives contribute positively while aligned with our objective of minimizing viscosity. The acquisition value and its gradient also estimated using Sobol‐QMC sampling as described for single‐objective optimization.

### 4.6 Implementation of SHaply Additive Explanations (SHAP)

Calculation and visualization of SHAP values was implemented using the SHAP python package (v0.48.0). The full dataset collected from all 72 formulations was used as background to determine the base SHAP value for each GPR model. As the GPR models were trained using scaled inputs and outputs, SHAP values were first transformed from scaled into real units prior to plotting. This was achieved by calculating the scale factor originally used to transform the output (via the MinMaxScaler function) and then multiplying SHAP values by this factor, thus preserving their additive nature. The inverse transformation of the MinMaxScaler was then used to change the SHAP base value into native measurement units.

## Author Contributions

**Elena Di Mare**: investigation, data curation, methodology. **D. Christopher Radford**: methodology, validation, software, data curation, supervision, investigation, writing – original draft, writing – review and editing. **Matthew Tamasi**: conceptualization, investigation, methodology, software. **Adam J. Gormley**: conceptualization, funding acquisition, project administration, supervision.

## Funding

This work was supported by funding from the National Institutes of Health (NIH) NIBIB R01EB037022 and NIGMS R35GM138296, as well as National Science Foundation (NSF) DMREF 2118860 and CBET 2309852.

## Conflicts of Interest

MT and AJG co‐founded Plexymer, Inc. which has licensed technology associated with this work. The authors declare no other competing financial interest.

## Supporting information




**Supporting File**: advs76551‐sup‐0001‐SuppMat.pdf.

## Data Availability

All model training data and programs comprising the active learning pipeline (including functions for data processing, model training, model validation, single‐ and multi‐objective Bayesian optimization, and exAI analysis) are available on GitHub at https://github.com/GormleyLab/AL‐for‐Bioformulation.

## References

[1] R.‐M. Lu , Y.‐C. Hwang , I. J. Liu , et al., “Development of Therapeutic Antibodies for the Treatment of Diseases,” Journal of Biomedical Science 27, no. 1 (2020): 1. 10.1186/s12929-019-0592-z PMC6939334 31894001

[2] L. Marks , “The Birth Pangs of Monoclonal Antibody Therapeutics,” mAbs 4, no. 3 (2014): 403–412, 10.4161/mabs.19909. PMC3355486 22531443

[3] S. Crescioli , H. Kaplon , L. Wang , J. Visweswaraiah , V. Kapoor , and J. M. Reichert , “Antibodies to Watch in 2025,” mAbs 17, no. 1 (2024): 2443538, 10.1080/19420862.2024.2443538. PMC12952251 39711140

[4] X. Lyu , Q. Zhao , J. Hui , et al., “The Global Landscape of Approved Antibody Therapies,” Antibody Therapeutics 5, no. 4 (2022): 233–257, 10.1093/abt/tbac021. PMC9535261 36213257

[5] P. Sharma , R. V. Joshi , R. Pritchard , K. Xu , and M. A. Eicher , “Therapeutic Antibodies in Medicine,” Molecules 28, no. 18 (2023): 6438. 10.3390/molecules28186438 PMC10535987 37764213

[6] V. Sifniotis , E. Cruz , B. Eroglu , and V. Kayser , “Current Advancements in Addressing Key Challenges of Therapeutic Antibody Design, Manufacture, and Formulation,” Antibodies 8, no. 2 (2019): 36, 10.3390/antib8020036. PMC6640721 31544842

[7] R. G. Strickley and W. J. Lambert , “A review of Formulations of Commercially Available Antibodies,” Journal of Pharmaceutical Sciences 110, no. 7 (2021): 2590–2608, 10.1016/j.xphs.2021.03.017. 33789155

[8] W. Wang , S. Singh , D. L. Zeng , K. King , and S. Nema , “Antibody Structure, Instability, and Formulation,” Journal of Pharmaceutical Sciences 96, no. 1 (2007): 1–26, 10.1002/jps.20727. 16998873

[9] K. D. Ratanji , J. P. Derrick , R. J. Dearman , and I. Kimber , “Immunogenicity of Therapeutic Proteins: Influence of Aggregation,” Journal of Immunotoxicology 11, no. 2 (2013): 99–109, 10.3109/1547691X.2013.821564. PMC4002659 23919460

[10] É. Kollár , B. Balázs , T. Tari , and I. Siró , “Development Challenges of High Concentration Monoclonal Antibody Formulations,” Drug Discovery Today: Technologies 37 (2020): 31–40, 10.1016/j.ddtec.2020.08.005. 34895653

[11] S. Wang , N. Zhang , T. Hu , et al., “Viscosity‐Lowering Effect of Amino Acids and Salts on Highly Concentrated Solutions of Two IgG1 Monoclonal Antibodies,” Molecular Pharmaceutics 12, no. 12 (2015): 4478–4487, 10.1021/acs.molpharmaceut.5b00643. 26528726

[12] J. J. Hung , B. J. Dear , C. A. Karouta , et al., “Protein–Protein Interactions of Highly Concentrated Monoclonal Antibody Solutions via Static Light Scattering and Influence on the Viscosity,” The Journal of Physical Chemistry B 123, no. 4 (2019): 739–755, 10.1021/acs.jpcb.8b09527. 30614707

[13] D. S. Tomar , S. Kumar , S. K. Singh , S. Goswami , and L. Li , “Molecular Basis of High Viscosity in Concentrated Antibody Solutions: Strategies for High Concentration Drug Product Development,” mAbs 8, no. 2 (2016): 216–228, 10.1080/19420862.2015.1128606. PMC5074600 26736022

[14] C. A. Mieczkowski , “The Evolution of Commercial Antibody Formulations,” Journal of Pharmaceutical Sciences 112, no. 7 (2023): 1801–1810, 10.1016/j.xphs.2023.03.026. 37037341

[15] I. AbbVie , Skyrizi (risankizumab‐rzaa) injection, for subcutaneous or intravenous use: Prescribing Information, (AbbVie Inc., 2026), accessed 2026/05/01, https://dailymed.nlm.nih.gov/dailymed/drugInfo.cfm?setid=7148c8eb‐b39e‐e20a‐6494‐a6df82392858.

[16] I. Regeneron Pharmaceuticals , Dupixent (dupilumab) injection, for subcutaneous use: Prescribing Information, (Regeneron Pharmaceuticals, Inc., 2025), accessed 2026/05/01, https://dailymed.nlm.nih.gov/dailymed/drugInfo.cfm?setid=595f437d‐2729‐40bb‐9c62‐c8ece1f82780.

[17] J. W. P. Zajac , P. Muralikrishnan , C. L. Heldt , S. L. Perry , and S. Sarupria , “Towards Stable Biologics: Understanding Co‐Excipient Effects on Hydrophobic Interactions and Solvent Network Integrity,” Molecular Systems Design & Engineering 10, no. 6 (2025): 432–446, 10.1039/D4ME00201F.

[18] E. Sahin , A. O. Grillo , M. D. Perkins , and C. J. Roberts , “Comparative Effects of pH and Ionic Strength on Protein–Protein Interactions, Unfolding, and Aggregation for IgG1 Antibodies,” Journal of Pharmaceutical Sciences 99, no. 12 (2010): 4830–4848, 10.1002/jps.22198. 20821389

[19] T. J. Kamerzell , R. Esfandiary , S. B. Joshi , C. R. Middaugh , and D. B. Volkin , “Protein–Excipient Interactions: Mechanisms and Biophysical Characterization Applied to Protein Formulation Development,” Advanced Drug Delivery Reviews 63, no. 13 (2011): 1118–1159, 10.1016/j.addr.2011.07.006. 21855584

[20] D. Yang and L. M. Walker , “Synergistic Effects of Multiple Excipients on Controlling Viscosity of Concentrated Protein Dispersions,” Journal of Pharmaceutical Sciences 112, no. 5 (2023): 1379–1387, 10.1016/j.xphs.2022.12.011. 36539064

[21] P.‐K. Lai , A. Fernando , T. K. Cloutier , et al., “Machine Learning Applied to Determine the Molecular Descriptors Responsible for the Viscosity Behavior of Concentrated Therapeutic Antibodies,” Molecular Pharmaceutics 18, no. 3 (2021): 1167–1175. 10.1021/acs.molpharmaceut.0c01073 33450157

[22] P.‐K. Lai , A. Gallegos , N. Mody , H. A. Sathish , and B. L. Trout , “Machine Learning Prediction of Antibody Aggregation and Viscosity for High Concentration Formulation Development of Protein Therapeutics,” mAbs 14, no. 1 (2022): 2026208, 10.1080/19420862.2022.2026208. PMC8794240 35075980

[23] T. Jain , T. Sun , S. Durand , et al., “Biophysical Properties of the Clinical‐Stage Antibody Landscape,” Proceedings of the National Academy of Sciences 114, no. 5 (2017): 944–949, 10.1073/pnas.1616408114. PMC5293111 28096333

[24] H. Shim , “Bispecific Antibodies and Antibody–Drug Conjugates for Cancer Therapy: Technological Considerations,” Biomolecules 10, no. 3 (2020): 360, 10.3390/biom10030360. PMC7175114 32111076

[25] J. Zarzar , T. Khan , M. Bhagawati , B. Weiche , J. Sydow‐Andersen , and A. Sreedhara , “High Concentration Formulation Developability Approaches and Considerations,” mAbs 15, no. 1 (2023): 2211185, 10.1080/19420862.2023.2211185. PMC10190182 37191233

[26] Z. Bao , J. Bufton , R. J. Hickman , A. Aspuru‐Guzik , P. Bannigan , and C. Allen , “Revolutionizing Drug Formulation Development: The Increasing Impact of Machine Learning,” Advanced Drug Delivery Reviews 202 (2023): 115108, 10.1016/j.addr.2023.115108. 37774977

[27] V. L. Deringer , A. P. Bartók , N. Bernstein , D. M. Wilkins , M. Ceriotti , and G. Csányi , “Gaussian Process Regression for Materials and Molecules,” Chemical Reviews 121, no. 16 (2021): 10073–10141, 10.1021/acs.chemrev.1c00022. PMC8391963 34398616

[28] B. Shahriari , K. Swersky , Z. Wang , R. P. Adams , and N. De Freitas , “Taking the Human Out of the Loop: A Review of Bayesian Optimization,” Proceedings of the IEEE 104, no. 1 (2015): 148–175, 10.1109/JPROC.2015.2494218.

[29] R. Rodríguez‐Pérez , F. Miljković , and J. Bajorath , “Machine Learning in Chemoinformatics and Medicinal Chemistry,” Annual Review of Biomedical Data Science 5, no. 1 (2022): 43–65. 10.1146/annurev-biodatasci-122120-124216 35440144

[30] J. Thompson , W. P. Walters , J. A. Feng , et al., “Optimizing Active Learning for Free Energy Calculations,” Artificial Intelligence in the Life Sciences 2 (2022): 100050, 10.1016/j.ailsci.2022.100050.

[31] J. Panteleev , H. Gao , and L. Jia , “Recent Applications of Machine Learning in Medicinal Chemistry,” Bioorganic & Medicinal Chemistry Letters 28, no. 17 (2018): 2807–2815, 10.1016/j.bmcl.2018.06.046. 30122222

[32] P. Suriyaamporn , B. Pamornpathomkul , P. Patrojanasophon , T. Ngawhirunpat , T. Rojanarata , and P. Opanasopit , “The Artificial Intelligence‐Powered New Era in Pharmaceutical Research and Development: A Review,” Aaps Pharmscitech [Electronic Resource] 25, no. 6 (2024): 188. 10.1208/s12249-024-02901-y 39147952

[33] A. Arslan , B. Yet , E. Nemutlu , Y. Akdağ Çaylı , H. Eroğlu , and L. Öner , “Celecoxib Nanoformulations With Enhanced Solubility, Dissolution Rate, and Oral Bioavailability: Experimental Approaches Over In Vitro/In Vivo Evaluation,” Pharmaceutics 15, no. 2 (2023): 363. 10.3390/pharmaceutics15020363 PMC9964073 36839685

[34] P. Bannigan , M. Aldeghi , Z. Bao , F. Häse , A. Aspuru‐Guzik , and C. Allen , “Machine Learning Directed Drug Formulation Development,” Advanced Drug Delivery Reviews 175 (2021): 113806. 10.1016/j.addr.2021.05.016 34019959

[35] H. Narayanan , J. A. Hinckley , R. Barry , et al., “Accelerating Cell Culture Media Development Using Bayesian Optimization‐Based Iterative Experimental Design,” Nature Communications 16, no. 1 (2025): 6055, 10.1038/s41467-025-61113-5. PMC12218302 40593792

[36] E. Claes , T. Heck , K. Coddens , M. Sonnaert , J. Schrooten , and J. Verwaeren , “Bayesian Cell Therapy Process Optimization,” Biotechnology and Bioengineering 121, no. 5 (2024): 1569–1582, 10.1002/bit.28669. 38372656

[37] J. Bader , H. Narayanan , P. Arosio , and J.‐C. Leroux , “Improving Extracellular Vesicles Production Through a Bayesian Optimization‐Based Experimental Design,” European Journal of Pharmaceutics and Biopharmaceutics 182 (2023): 103–114, 10.1016/j.ejpb.2022.12.004. 36526027

[38] A. Khan , A. I. Cowen‐Rivers , A. Grosnit , et al., “Toward Real‐World Automated Antibody Design With Combinatorial Bayesian Optimization,” Cell Reports Methods 3, no. 1 (2023): 100374, 10.1016/j.crmeth.2022.100374. PMC9939385 36814835

[39] G. Schneider , “Automating Drug Discovery,” Nature Reviews Drug Discovery 17, no. 2 (2017): 97–113, 10.1038/nrd.2017.232. 29242609

[40] A. J. Oyejide , Y. A. Adekunle , O. D. Abodunrin , and E. O. Atoyebi , “Artificial Intelligence, Computational Tools and Robotics for Drug Discovery, Development, and Delivery,” Intelligent Pharmacy 3, no. 3 (2025): 207–224, 10.1016/j.ipha.2025.01.001.

[41] G. Tom , S. P. Schmid , S. G. Baird , et al., “Self‐Driving Laboratories for Chemistry and Materials Science,” Chemical Reviews 124, no. 16 (2024): 9633–9732, 10.1021/acs.chemrev.4c00055. PMC11363023 39137296

[42] E. O. Pyzer‐Knapp , J. W. Pitera , P. W. J. Staar , et al., “Accelerating Materials Discovery Using Artificial Intelligence, High Performance Computing and Robotics,” npj Computational Materials 8, no. 1 (2022): 84.

[43] S. Kosuri , C. H. Borca , H. Mugnier , et al., “Machine‐Assisted Discovery of Chondroitinase ABC Complexes Toward Sustained Neural Regeneration,” Advanced Healthcare Materials 11, no. 10 (2022): 2102101. 10.1002/adhm.202102101 PMC9119153 35112508

[44] M. J. Tamasi , R. A. Patel , C. H. Borca , et al., “Machine Learning on a Robotic Platform for the Design of Polymer–Protein Hybrids,” Advanced Materials 34, no. 30 (2022): 2201809, 10.1002/adma.202201809. PMC9339531 35593444

[45] E. J. Di Mare , A. Punia , M. S. Lamm , T. A. Rhodes , and A. J. Gormley , “Data‐Driven Design of Novel Polymer Excipients for Pharmaceutical Amorphous Solid Dispersions,” Bioconjugate Chemistry 35, no. 9 (2024): 1363–1372. 10.1021/acs.bioconjchem.4c00294 PMC13054844 39150455

[46] H. Narayanan , F. Dingfelder , I. Condado Morales , et al., “Design of Biopharmaceutical Formulations Accelerated by Machine Learning,” Molecular Pharmaceutics 18, no. 10 (2021): 3843–3853, 10.1021/acs.molpharmaceut.1c00469. 34519511

[47] I. Waibel , T. N. Schneider , F. J. Fischer , et al., “Bayesian Optimization for Efficient Multiobjective Formulation Development of Biologics,” Molecular Pharmaceutics 22, no. 11 (2025): 6636–6645, 10.1021/acs.molpharmaceut.5c00591. PMC12587402 41002022

[48] F. He , G. W. Becker , J. R. Litowski , L. O. Narhi , D. N. Brems , and V. I. Razinkov , “High‐Throughput Dynamic Light Scattering Method for Measuring Viscosity of Concentrated Protein Solutions,” Analytical Biochemistry 399, no. 1 (2010): 141–143, 10.1016/j.ab.2009.12.003. 19995543

[49] M. Prašnikar , M. Bjelošević Žiberna , N. Kržišnik , et al., “Additive Effects of the New Viscosity‐Reducing and Stabilizing Excipients for Monoclonal Antibody Formulation,” International Journal of Pharmaceutics 674 (2025): 125451. 10.1016/j.ijpharm.2025.125451 40064383

[50] J. Wen , H. Lord , N. Knutson , and M. Wikström , “Nano Differential Scanning Fluorimetry for Comparability Studies of Therapeutic Proteins,” Analytical Biochemistry 593 (2020): 113581. 10.1016/j.ab.2020.113581 31935356

[51] F. He , S. Hogan , R. F. Latypov , L. O. Narhi , and V. I. Razinkov , “High Throughput Thermostability Screening of Monoclonal Antibody Formulations,” Journal of Pharmaceutical Sciences 99, no. 4 (2010): 1707–1720, 10.1002/jps.21955. 19780136

[52] K. Dauer , S. Pfeiffer‐Marek , W. Kamm , and K. G. Wagner , “Microwell Plate‐Based Dynamic Light Scattering as a High‐Throughput Characterization Tool in Biopharmaceutical Development,” Pharmaceutics 13, no. 2 (2021): 172, 10.3390/pharmaceutics13020172. PMC7911513 33514069

[53] A. Y. Xu , M. A. Blanco , M. M. Castellanos , et al., “Role of Domain–Domain Interactions on the Self‐Association and Physical Stability of Monoclonal Antibodies: Effect of pH and Salt,” The Journal of Physical Chemistry B 127, no. 39 (2023): 8344–8357, 10.1021/acs.jpcb.3c03928. PMC10561141 37751332

[54] M. J. Tamasi and A. J. Gormley , “Biologic Formulation in a Self‐Driving Biomaterials Lab,” Cell Reports Physical Science 3, no. 9 (2022): 101041, 10.1016/j.xcrp.2022.101041.

[55] E. J. Braham , R. D. Davidson , M. Al‐Hashimi , R. Arroyave , and S. Banerjee , “Navigating the Design Space of Inorganic Materials Synthesis Using Statistical Methods and Machine Learning,” Dalton Transactions 49, no. 33 (2020): 11480–11488. 10.1039/d0dt02028a 32743629

[56] W.‐L. Loh , “On Latin Hypercube Sampling,” The Annals of Statistics 24, no. 5 (1996): 2058–2080.

[57] E. Schulz , M. Speekenbrink , and A. Krause , “A Tutorial on Gaussian Process Regression: Modelling, Exploring, and Exploiting Functions,” Journal of Mathematical Psychology 85 (2018): 1–16, 10.1016/j.jmp.2018.03.001.

[58] M. Balandat , B. Karrer , D. Jiang , et al., “BoTorch: A Framework for Efficient Monte‐Carlo Bayesian Optimization,” in Advances in Neural Information Processing Systems (Curran Associates, Inc., 2020), 21524–21538.

[59] N. Srinivas , A. Krause , S. M. Kakade , and M. W. Seeger , “Information‐Theoretic Regret Bounds for Gaussian Process Optimization in the Bandit Setting,” IEEE Transactions on Information Theory 58, no. 5 (2012): 3250–3265, 10.1109/TIT.2011.2182033.

[60] D. Ginsbourger , R. Le Riche , and L. Carraro , “Kriging Is Well‐Suited to Parallelize Optimization,” in Computational Intelligence in Expensive Optimization Problems, Edited by Y. Tenne and C.‐K. Goh (Springer, 2010), 131–162.

[61] M. T. Ribeiro , S. Singh , and C. Guestrin , “Why Should I Trust You?″: Explaining the Predictions of Any Classifier,” in Proceedings of the 22nd ACM SIGKDD International Conference on Knowledge Discovery and Data Mining (Association for Computing Machinery, 2016), 1135–1144, 10.1145/2939672.2939778.

[62] S. M. Lundberg and S.‐I. Lee , “A Unified Approach to Interpreting Model Predictions,” in Advances in Neural Information Processing Systems (Curran Associates, Inc., 2017), 4765–4774.

[63] S. M. Lundberg , G. Erion , H. Chen , et al., “From Local Explanations to Global Understanding With Explainable AI for Trees,” Nature Machine Intelligence 2, no. 1 (2020): 56–67, 10.1038/s42256-019-0138-9. PMC7326367 32607472

[64] H. L. Svilenov , A. Kulakova , M. Zalar , A. P. Golovanov , P. Harris , and G. Winter , “Orthogonal Techniques to Study the Effect of pH, Sucrose, and Arginine Salts on Monoclonal Antibody Physical Stability and Aggregation During Long‐Term Storage,” Journal of Pharmaceutical Sciences 109, no. 1 (2020): 584–594, 10.1016/j.xphs.2019.10.065. 31689429

[65] S. Ren , “Effects of Arginine in Therapeutic Protein Formulations: A Decade Review and Perspectives,” Antibody Therapeutics 6, no. 4 (2023): 265–276, 10.1093/abt/tbad022. PMC10702853 38075239

[66] B. K. Meyer , B. Hu , R. Ionescu , et al., “Opalescence of an IgG1 Monoclonal Antibody Formulation Mediated by Ionic Strength and Excipients,” BioPharm International 22, no. 4 (2022): 36–47.

[67] J. H. Friedman and B. E. Popescu , “Predictive Learning via Rule Ensembles,” The Annals of Applied Statistics 2, no. 3 (2008): 916–954, 10.1214/07-AOAS148.

[68] E. Hüllermeier and W. Waegeman , “Aleatoric and Epistemic Uncertainty in Machine Learning: An Introduction to Concepts and Methods,” Machine learning 110, no. 3 (2021): 457–506, 10.1007/s10994-021-05946-3.

[69] T. K. Cloutier , C. Sudrik , N. Mody , H. A. Sathish , and B. L. Trout , “Machine Learning Models of Antibody–Excipient Preferential Interactions for Use in Computational Formulation Design,” Molecular Pharmaceutics 17, no. 9 (2020): 3589–3599. 10.1021/acs.molpharmaceut.0c00629 32794710

[70] L. Li , S.‐I. Back , J. Ma , Y. Guo , T. Galeandro‐Diamant , and D. Clénet , “Bayesian Optimization and Machine Learning for Vaccine Formulation Development,” PLoS One 20, no. 6 (2025): 0324205. 10.1371/journal.pone.0324205 PMC12157168 40498693

[71] N. Whitaker , J. Xiong , S. E. Pace , et al., “A Formulation Development Approach to Identify and Select Stable Ultra–High‐Concentration Monoclonal Antibody Formulations With Reduced Viscosities,” Journal of Pharmaceutical Sciences 106, no. 11 (2017): 3230–3241, 10.1016/j.xphs.2017.06.017. 28668340

[72] D. Baizhigitova , I. E. Wu , L. Kalejaye , and P.‐K. Lai , “Molecular Modeling and Machine Learning for Predicting High‐Concentration Antibody Viscosity,” Advanced Drug Delivery Reviews 233 (2026): 115839. 10.1016/j.addr.2026.115839 41780732

[73] E. K. Makowski , T. Wang , J. M. Zupancic , et al., “Optimization of Therapeutic Antibodies for Reduced Self‐Association and Non‐Specific Binding via Interpretable Machine Learning,” Nature Biomedical Engineering 8, no. 1 (2023): 45–56, 10.1038/s41551-023-01074-6. PMC10842909 37666923

[74] K. Huynh and C. L. Partch , “Analysis of Protein Stability and Ligand Interactions by Thermal Shift Assay,” Current Protocols in Protein Science 79, no. 1 (2015): 28–39. 10.1002/0471140864.ps2809s79 PMC4332540 25640896

[75] A. A. Bhirde , M.‐J. Chiang , R. Venna , S. Beaucage , and K. Brorson , “High‐Throughput in‐use and Stress Size Stability Screening of Protein Therapeutics Using Algorithm‐Driven Dynamic Light Scattering,” Journal of Pharmaceutical Sciences 107, no. 8 (2018): 2055–2062, 10.1016/j.xphs.2018.04.017. 29715479

[76] J. Dick and F. Pillichshammer , Digital Nets and Sequences: Discrepancy Theory and Quasi‐Monte Carlo Integration (Cambridge University Press, 2010), 10.1017/CBO9780511761188.

[77] I. M. Sobol , “On the Distribution of Points in a Cube and the Approximate Evaluation of Integrals,” USSR Computational Mathematics and Mathematical Physics 7, no. 4 (1967): 86–112.

