# Supplementary material for: Automation and Active Learning for the Multi‐Objective Optimization of Antibody Formulations
Source: Adv Sci (Weinh). 2026 Jul 13:e76551. Online ahead of print. doi: 10.1002/advs.76551 (PMC13360116; doi:10.1002/advs.76551)
Supplement: Supplementary file 1 — Supporting File: advs76551‐sup‐0001‐SuppMat.pdf. [file ADVS-9999-e76551-s001.pdf]

Supplementary Information

Automation and Active Learning for the Multi-Objective Optimization of Antibody Formulations

D. Christopher Radford*, Matthew Tamasi*, Elena Di Mare, Adam J. Gormley

Department of Biomedical Engineering, Rutgers, The State University of New Jersey, Piscataway, New Jersey 08854, USA

Corresponding Author: adam.gormley@rutgers.edu

Note: All model training data and programs comprising the active learning pipeline (including functions for data processing, model training, model validation, single- and multi-objective Bayesian optimization, and exAI analysis) are available on GitHub at https://github.com/GormleyLab/AL-for-Bioformulation.

Table S1. *Design space for antibody formulation*

Additive	Class	Concentration Range
Sodium Acetate Buffer: (pH 4.0, 4.5, 5.0, 5.5)	Buffer	5 – 100mM
Citrate-Phosphate Buffer: (pH 4.0, 5.0, 6.0, 7.0)	Buffer	5 – 100mM
L-Arginine	Amino Acid	1 – 125 mM
Sucrose	Sugar	0 – 2.5 wt%
NaCl	Salt	0 – 150 mM

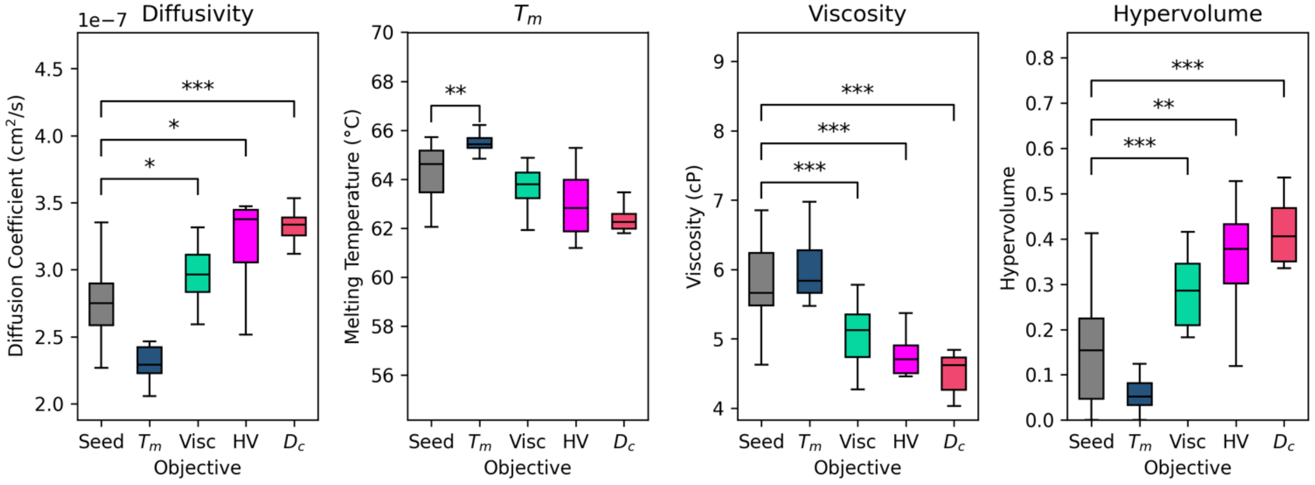

Figure S1. Measurements acquired for formulation diffusivity, T_m, viscosity, and hypervolume from combined active learning rounds (Gen 1 & Gen 2), organized by objective arm. Statistical significance was assessed between measured values for seed formulations and ML proposed formulations and determined by single tailed t-test. *($p < 0.05$), **($p < 0.005$), ***($p < 0.0005$), unlabeled pairs are not significantly different.

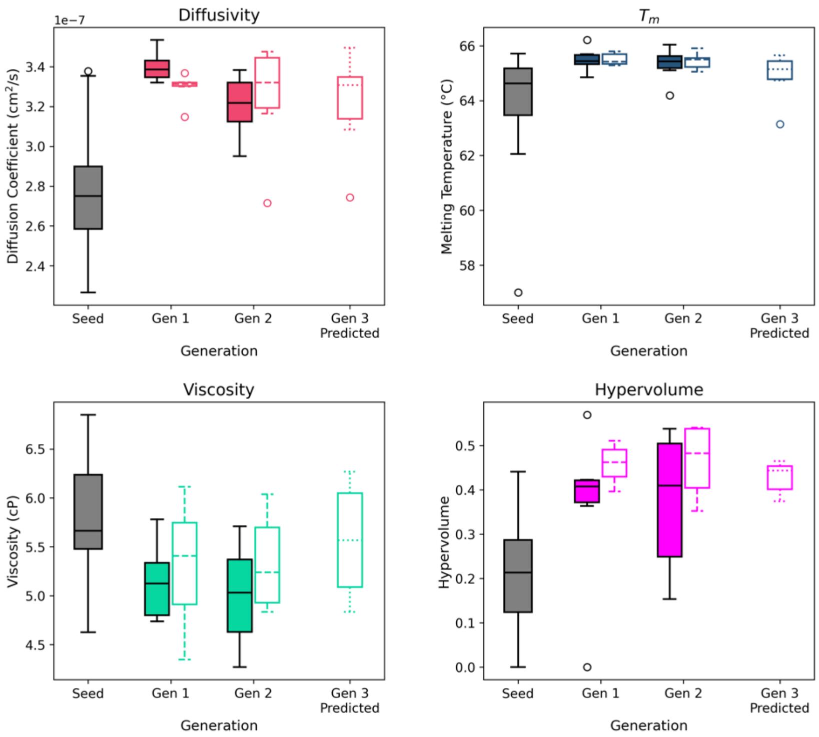

Figure S2. Extending forward-looking predictions from Fig. 2A to a hypothetical third generation of formulation candidates. Predictions for Gen 3 made from GPR models trained on all data collected through Generation 2 (72 total formulations). Proposed candidates for the hypothetical third generation are not expected to further improve the target properties over previous active learning generations.

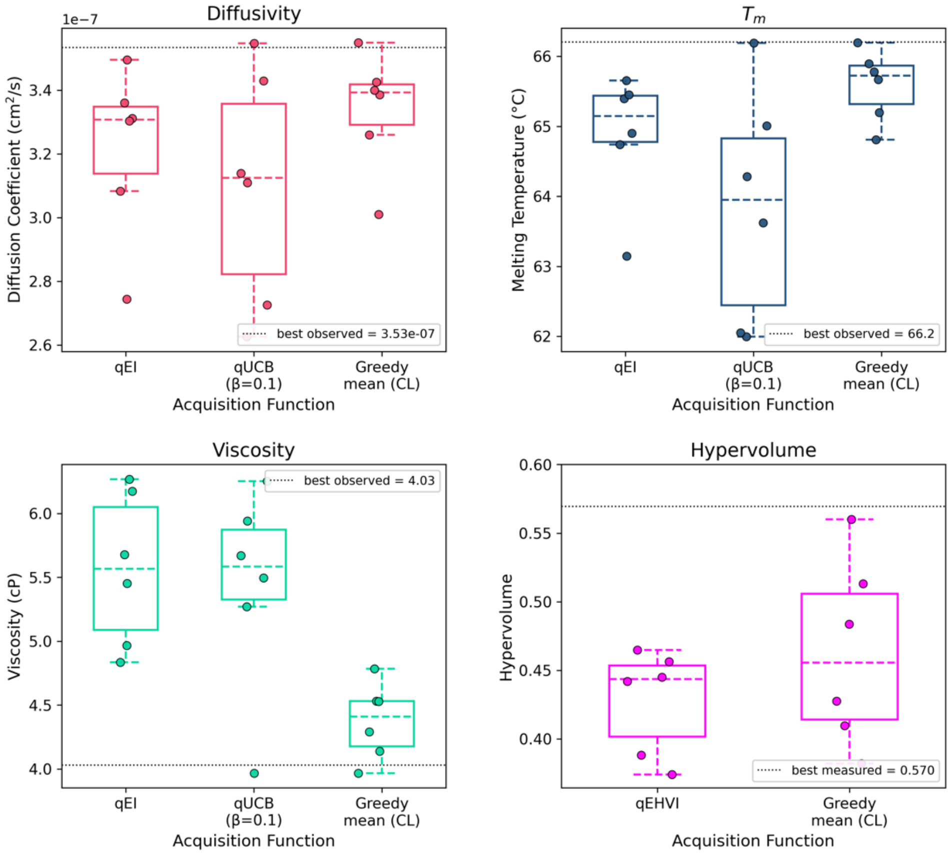

Figure S3. Hypothetical Gen 3 candidate distributions under alternative acquisition functions. Predicted Gen 3 candidate values for the three single-objective arms (Diffusivity, T_m, Viscosity) and the multi-objective hypervolume arm (HV). Predictions for Gen 3 were made from GPR models trained on all data collected through Generation 2 (72 total formulations). Baseline qEI/qEHVI acquisition functions are compared against more exploitative acquisition functions. The qEI/qEHVI candidates shown are the same hypothetical Gen 3 batch in Figure S2. qUCB (β = 0.1): q-upper confidence bound with a small exploration weight ($\mu + \sqrt{(\beta\pi/2)} \cdot \sigma$), strongly biased toward exploitation while retaining a slight uncertainty term. Greedy mean (CL): Constant-liar batching scheme (CL-min variant) for pure-exploitation acquisition with no uncertainty term in the score. Lead candidates from all acquisition functions are predicted to offer minimal (<2%) improvement over the best-observed formulation already in the library (dotted line), indicating that the Gen 2 plateau reflects an intrinsic limit of the design space rather than a consequence of qEI/qEHVI's explore-exploit balance.

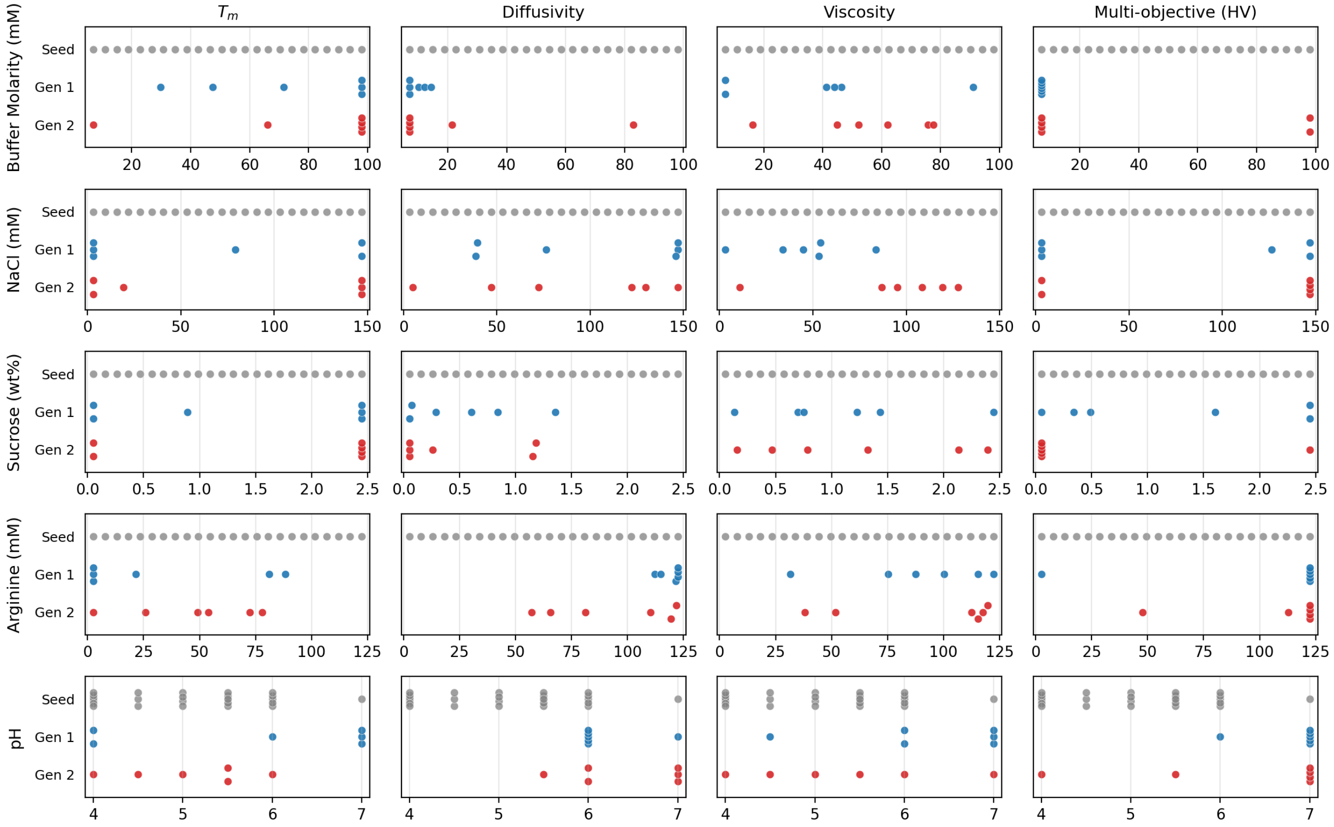

Figure S4: Per-arm feature distributions across the active learning campaign. Individual values of each formulation feature (buffer molarity, NaCl, sucrose, arginine, and pH) for the 24 seed formulations (grey) and the six candidates proposed by each of the four optimization arms in Gen 1 (blue) and Gen 2 (red). Columns correspond to the T_m, diffusion, viscosity, and multi-objective hypervolume (HV) optimization arms; rows correspond to the five continuous formulation features.

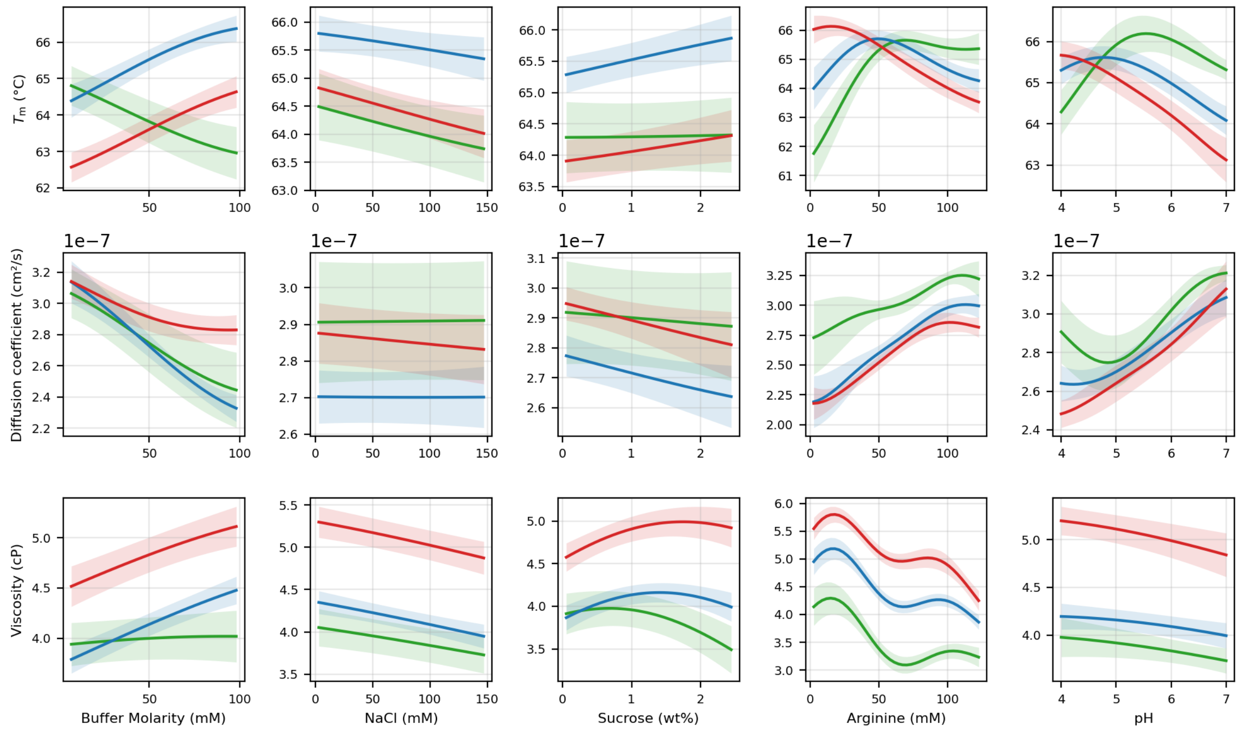

Figure S5. One-dimensional GPR partial-dependence slices for each formulation feature against each modeled property. Each curve sweeps the indicated feature across its observed range while every other continuous variable is held at the 25th (green), 50th (blue), or 75th (red) percentile of its range. Buffer is fixed to citrate-phosphate at all three anchors and pH is set to 4.0 / 5.0 / 6.0 (green / blue / red), so each curve corresponds to a chemically valid buffer/pH pair rather than interpolating between buffer systems. Shaded bands show ±1 SD of the GPR posterior.

Table S2. *Additive linear surrogate fit and interaction fraction*

Objective	Linear surrogate R^2	Main-effect fraction	Interaction fraction
T_m	0.22	49%	51%
Diffusivity	0.82	83%	17%
Viscosity	0.79	88%	12%

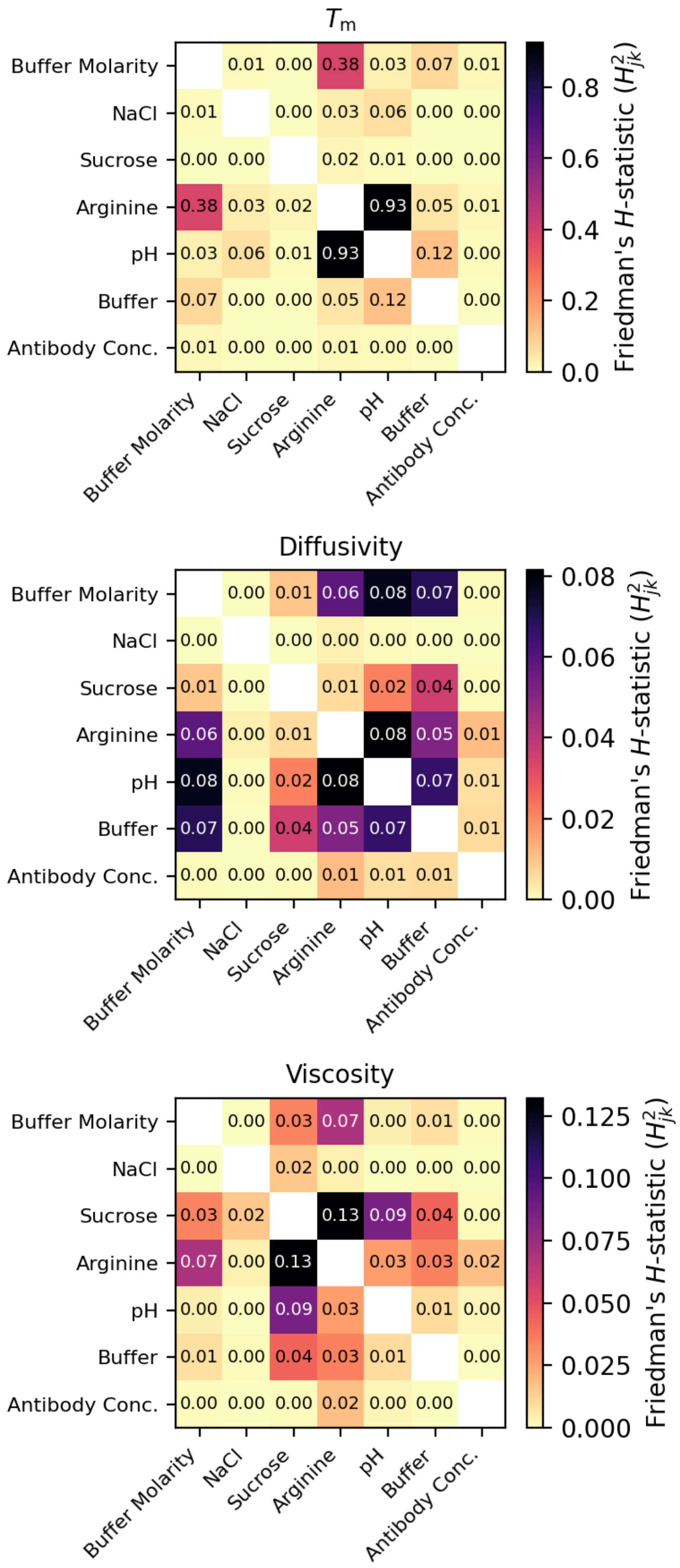

Figure S6. Pairwise interaction strength via Friedman's H-statistic for each GPR model. Friedman's H-statistic ($\mathrm{H}_{\mathrm{jk}}^2$) is a model-agnostic measure of pairwise interaction strength built on partial dependence functions. It measures the fraction of the joint partial dependence of two features that cannot be explained by the sum of their individual partial dependence functions, with 0 indicating purely additive behavior and higher values indicating stronger pairwise interaction. The diffusivity and viscosity models show limited pairwise interactions between formulation features, while the T_m model exhibits strong interactions between arginine concentration and the buffer conditions (both buffer pH and molarity). This indicates that thermal stability is shaped by non-additive coupling between excipients to a greater extent than the other formulation properties.

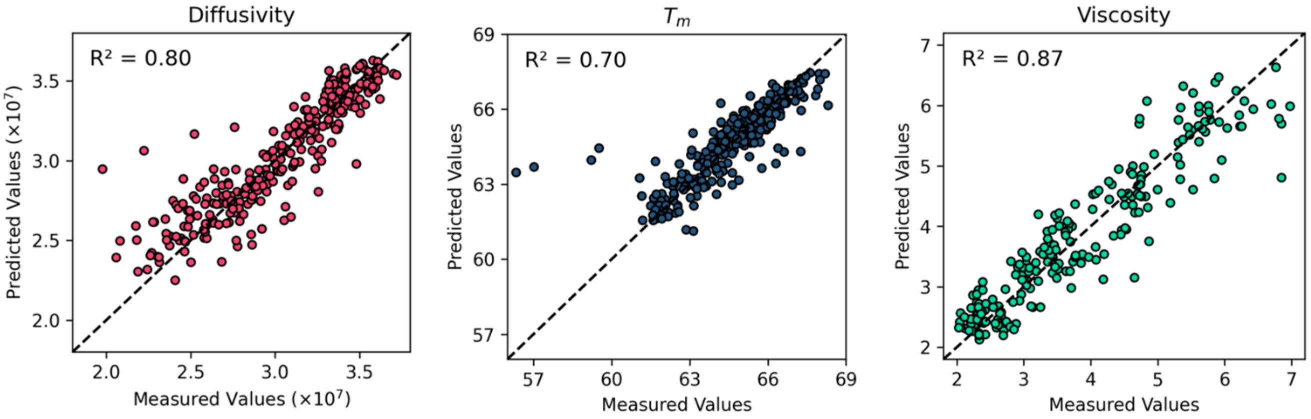

Figure S7. Trained GPR surrogate model accuracy across all 72 formulations, using group k-fold cross validation (10 folds) for diffusivity, T_m, viscosity respectively.

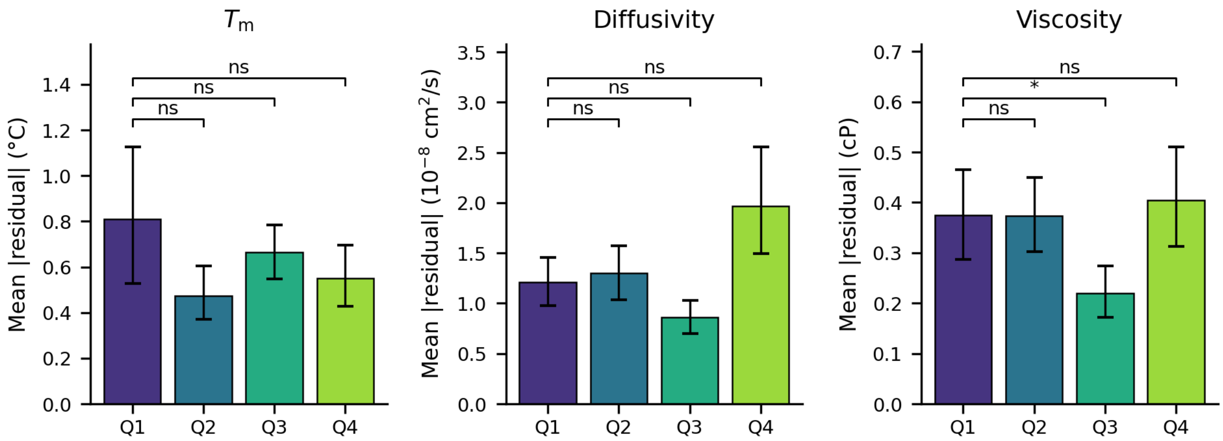

Figure S8. Out-of-sample mean absolute residual stratified by similarity to the training set. For each held-out prediction from the 10-fold *GroupKFold* cross-validation, the Euclidean distance to its nearest training-set neighbor was computed in the 7-dimensional MinMax-scaled feature space. These distances were used to assign test points to one of four equal-frequency quartiles by their per-objective distance (Q1 = nearest, Q4 = farthest). Bars show the per-bin mean |residual| in real units; error bars are the 95% confidence interval from a 1000-iteration percentile bootstrap. The Q1 residual distribution was compared pairwise against each of Q2, Q3, and Q4 using two-sided Mann-Whitney U tests; reported significance reflects Holm-Bonferroni correction across the three pairwise comparisons per objective (ns: $p \geq 0.05$; *: $p < 0.05$). The data suggest that predictive performance was broadly maintained regardless of formulation novelty relative to the training set.

Table S3: *Summary Statistics of Gen-on-Gen Uncertainty Improvement*

Objective	Training Data	Statistical Metric				
		**Mean**	**Median**	**Min**	**Max**	**IQR**
T_m (°C)	Seed Only	1.30	1.34	0.34	1.79	0.34
	Seed + Gen 1	0.84	0.84	0.26	1.50	0.28
	Seed + Gen 1/2	0.66	0.65	0.23	1.31	0.20
Diffusivity (cm^2/s)	Seed Only	2.09E-08	2.14E-08	5.75E-09	2.83E-08	5.57E-09
	Seed + Gen 1	1.74E-08	1.74E-08	3.84E-09	3.15E-08	6.08E-09
	Seed + Gen 1/2	1.36E-08	1.33E-08	3.70E-09	2.72E-08	4.57E-09
Viscosity (cP)	Seed Only	0.42	0.42	0.16	0.72	0.15
	Seed + Gen 1	0.28	0.28	0.12	0.50	0.08
	Seed + Gen 1/2	0.30	0.29	0.11	0.58	0.09

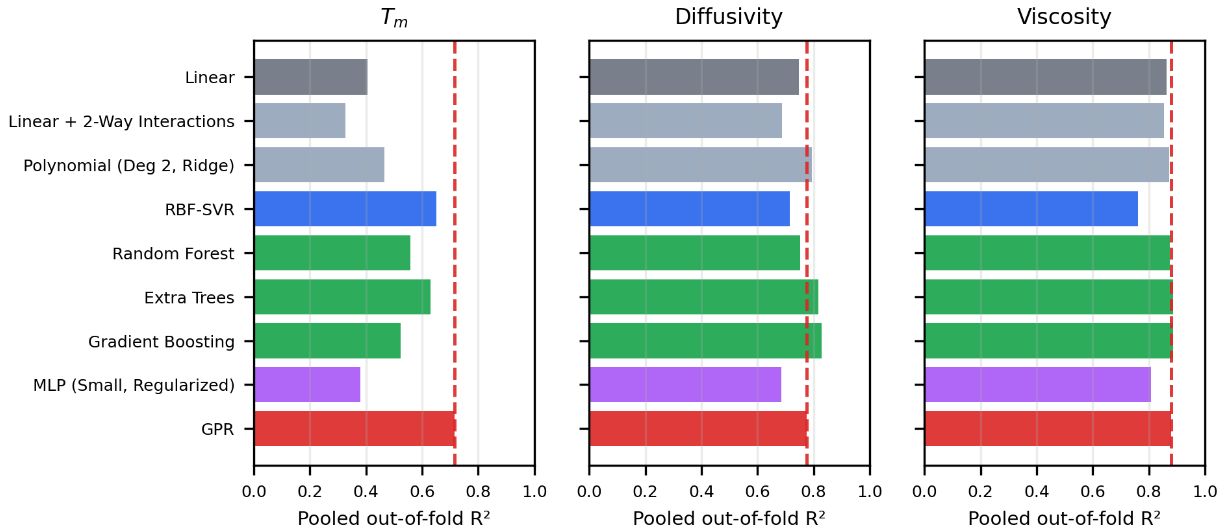

Figure S9. Expanded model-architecture benchmark on the full 72-formulation library. Pooled out-of-fold coefficient of determination (R^2) for nine regression architectures evaluated under matched 10-fold GroupKFold cross-validation grouped by Formulation ID, so that all concentration replicates of a given formulation were withheld together. The architectures span six model families. Linear: ordinary least-squares regression on the seven design features (buffer molarity, NaCl, sucrose, arginine, pH, buffer system, and antibody concentration). Linear + 2-Way Interactions: ordinary least-squares regression on the same seven features augmented with all 21 pairwise interaction terms (PolynomialFeatures(degree=2, interaction_only=True)). Polynomial (Deg 2, Ridge): ridge regression (α = 1.0) on the full degree-2 polynomial basis including main-effect, pairwise-interaction, and squared terms. RBF-SVR: ε-insensitive support-vector regression with a radial-basis-function kernel (C = 10, ε = 0.03, γ = "scale"). Random Forest: bootstrap-aggregated regression forest (300 trees, min_samples_leaf = 2). Extra Trees: extremely-randomized regression trees trained on the full dataset with random split thresholds (300 trees, min_samples_leaf = 2). Gradient Boosting: gradient-boosted decision trees (200 estimators, learning rate 0.035, maximum depth 2, min_samples_leaf = 3). MLP (Small, Regularized): a small two-layer feed-forward neural network (16 → 8 ReLU units, L_2 weight penalty α = 0.01, Adam optimizer, learning rate 2×10^{-3}, up to 3000 epochs with early stopping). GPR: Gaussian process regressor, same hyperparameters as used for BO campaign (see Section 4 of manuscript). Bar colors group architectures by model family; the dashed red reference line marks the GPR's pooled R^2 in each panel. The GPR showed the strongest performance for T_m, while several deterministic nonlinear models performed comparably for diffusivity and viscosity after the full active-learning dataset had been collected.

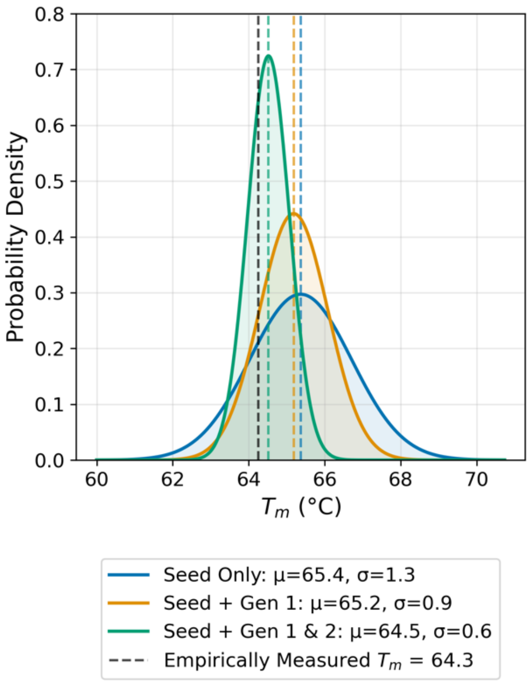

Figure S10. Representative prediction probability density function (PDF) from GPR surrogate model on formulation thermal stability trained on Seed data, Seed + Gen 1, and Seed + Gen 1 & Gen 2 data. Formulation being predicted is composed of: 15 mg/mL antibody in sodium acetate buffer (85.1 mM, pH 5.0) with 0.51 wt% sucrose, 54.7 mM arginine, and 62.8 mM NaCl. Note that this formulation is not part of any generation in the training data set.
